# α-Synuclein binds to the ER–mitochondria tethering protein VAPB to disrupt Ca^2+^ homeostasis and mitochondrial ATP production

**DOI:** 10.1007/s00401-017-1704-z

**Published:** 2017-03-23

**Authors:** Sébastien Paillusson, Patricia Gomez-Suaga, Radu Stoica, Daniel Little, Paul Gissen, Michael J. Devine, Wendy Noble, Diane P. Hanger, Christopher C. J. Miller

**Affiliations:** 1 Department of Basic and Clinical Neurosciences, Institute of Psychiatry, Psychology and Neuroscience, King’s College London, 5 Cutcombe road, London, SE5 9RX UK; 2 MRC Laboratory of Molecular Cell Biology, University College London, London, UK; 3 Department of Neuroscience, Physiology and Pharmacology, University College London, London, UK

**Keywords:** α-Synuclein, Endoplasmic reticulum, Mitochondria, Axonal transport, Calcium signaling, Autophagy

## Abstract

α-Synuclein is strongly linked to Parkinson’s disease but the molecular targets for its toxicity are not fully clear. However, many neuronal functions damaged in Parkinson’s disease are regulated by signalling between the endoplasmic reticulum (ER) and mitochondria. This signalling involves close physical associations between the two organelles that are mediated by binding of the integral ER protein vesicle-associated membrane protein-associated protein B (VAPB) to the outer mitochondrial membrane protein, protein tyrosine phosphatase-interacting protein 51 (PTPIP51). VAPB and PTPIP51 thus act as a scaffold to tether the two organelles. Here we show that α-synuclein binds to VAPB and that overexpression of wild-type and familial Parkinson’s disease mutant α-synuclein disrupt the VAPB-PTPIP51 tethers to loosen ER–mitochondria associations. This disruption to the VAPB-PTPIP51 tethers is also seen in neurons derived from induced pluripotent stem cells from familial Parkinson’s disease patients harbouring pathogenic triplication of the α-synuclein gene. We also show that the α-synuclein induced loosening of ER–mitochondria contacts is accompanied by disruption to Ca^2+^ exchange between the two organelles and mitochondrial ATP production. Such disruptions are likely to be particularly damaging to neurons that are heavily dependent on correct Ca^2+^ signaling and ATP.

**Electronic supplementary material:**

The online version of this article (doi:10.1007/s00401-017-1704-z) contains supplementary material, which is available to authorized users.

## Introduction

Parkinson’s disease is the second most common human neurodegenerative disease and is characterised by the preferential loss of dopaminergic neurons in the substantia nigra. The cell and molecular events that give rise to this neuronal loss are not properly understood but a number of lines of evidence suggest that abnormalities in α-synuclein are central to the disease process. First, mutations within the gene encoding α-synuclein and increased α-synuclein gene dosage involving duplication and triplication events cause familial, dominantly inherited forms of the disease [1, 12, 41, 45, 49, 66, 74, 92]. Second, α-synuclein is the major protein constituent of Lewy bodies and Lewy neurites which are hallmark pathologies of Parkinson’s disease [76, 80]. Finally, overexpression of wild-type and familial mutant α-synuclein can induce aspects of disease in transgenic mice [6, 7, 18].

Despite this evidence, the mechanisms by which altered α-synuclein metabolism might cause disease are not properly understood. α-Synuclein is a 140 amino acid protein of unclear function that is enriched in synapses and perinuclear regions of neurons. Within neurons, α-synuclein localizes to cytosolic and membrane compartments including synaptic vesicles, mitochondria and the endoplasmic reticulum (ER) (see reviews [32, 33, 91]). Recent evidence suggests that its membrane localization involves targeting to lipid rafts (also known as detergent resistant membranes; DRM) [31].

Overexpression of wild-type or familial mutant α-synuclein has been shown to damage a number of physiological processes. These include Ca^2+^ homeostasis [11, 38], lipid metabolism [9, 31], mitochondria including mitochondrial transport and biogenesis [22, 32, 60, 90, 91], the ER [59] and autophagy [88]. Indeed, the difficulty in deciphering α-synuclein toxicity is linking these apparently diverse pathological changes to a common disease pathway.

One area of cell physiology that impacts upon all of these features involves signalling between ER and mitochondria. Approximately 5–20% of the mitochondrial surface is closely apposed (10–30 nm distances) to ER membranes; these specialized regions of ER are termed mitochondria-associated ER membranes (MAM). A large body of evidence demonstrates that mitochondria communicate directly with ER through MAM to regulate a number of fundamental cellular processes. These include Ca^2+^ homeostasis, lipid metabolism, mitochondrial ATP production, mitochondrial transport and biogenesis, ER stress and the unfolded protein response (UPR) and autophagy (see reviews [46, 48, 63, 70, 82, 83]). Any α-synuclein-induced damage to ER–mitochondria associations thus represents a plausible route for explaining many features of Parkinson’s disease.

The mechanisms by which regions of ER are recruited to mitochondria are not fully known but electron microscopy (EM) studies reveal the presence of structures that appear to tether the two organelles [16]. Recently, the integral ER protein VAPB was shown to bind to the outer mitochondrial membrane protein PTPIP51 to form at least some of these tethers [20, 26, 40, 78, 79]. Thus, modulating VAPB and/or PTPIP51 expression induces appropriate changes in ER–mitochondria contacts and Ca^2+^ exchange between the two organelles which is a physiological readout of ER–mitochondria associations [20, 78]. Here, we show that α-synuclein binds to VAPB, disrupts the VAPB-PTPIP51 interaction and perturbs ER–mitochondria associations. We also confirm that a proportion of α-synuclein is present in MAM. Moreover, we demonstrate that α-synuclein-induced loosening of ER–mitochondria contacts affects Ca^2+^ exchange between the two organelles. As such, our findings reveal a new molecular mechanism to link α-synuclein and Parkinson’s disease.

## Materials and methods

### Plasmids and siRNAs

Mammalian expression vectors for human myc-tagged VAPB and haemagglutinin (HA)-tagged PTPIP51 in pCIneo, pCIneo control vector expressing *Escherichia coli* chloramphenicol acetyltransferase (CAT), wild-type α-synuclein, α-synucleinA53T and α-synucleinA30P in pcDNA3.1(−) and enhanced green fluorescent protein (EGFP) tagged versions in pEGFPC1, and AT1.03 cytosolic ATeam FRET based ATP reporter was all as described and [20, 34, 42, 71]. For the production of stable cell lines, wild-type and mutant untagged α-synuclein cDNA were cloned as *Nhe*I–*Hind*III fragments in pcDNA6.V5-His with a stop codon prior to the His tag. GST-VAPB and pRK172 α-synuclein *E. coli* expression vectors were as described [43, 78]. Control and human α-synuclein siRNAs were from Santa Cruz Biotechnology (sc-37007 and sc-29619, respectively).

### Antibodies

Rabbit and rat antibodies to VAPB and PTPIP51 were as described [20]. Mouse anti-glyceraldehyde 3-phosphate dehydrogenase (GAPDH), mouse anti-hemagglutinin (HA), mouse anti-myc 9B11 and rabbit anti-glycogen synthase kinase-3β (GSK3β) phosphorylated on serine-9 (inactive GSK3β) were from Cell Signalling. Rabbit anti-mitofusin-2, rabbit anti-HA, mouse anti-heat shock protein-60 (HSP60) and mouse anti-neurofilament heavy-chain (NFH) (N52) were from Sigma. Rabbit (sc-7011) and mouse (211) anti-α-synuclein, rabbit anti-translocase of the outer mitochondrial membrane protein-20 (TOM20), rabbit anti-fatty acid coenzyme A ligase long-chain 4 (FACL4) and mouse anti-Sigma-1 receptor were from Santa Cruz Biotechnology. Mouse anti-α-synuclein and mouse anti-calnexin were from BD Biosciences. Rabbit anti-EGFP and mouse anti-β-actin were from Abcam. Rabbit anti-PTPIP51 and chicken anti-MAP2 were from Genetex. Mouse anti-protein disulphide isomerase (PDI) was from Affinity Bioreagents, rabbit anti-myc was from Upstate and rabbit anti-GSK3β was from StressGen.

### Cell culture and transfection

SH-SY5Y and HEK293 cells were purchased from the European Collection of Cell Cultures. Cells and were maintained in Dulbecco’s modified Eagle’s medium (DMEM) containing 4.5 g/l glucose (HEK293 cells) or DMEM/F-12 (1:1) containing 3.15 g/glucose (SH-SY5Y cells) supplemented with 10% fetal bovine serum (Sera Laboratories), 2 mM l-glutamine, 1 mM sodium pyruvate, 100 IU/ml penicillin and 100 μg/ml streptomycin (Invitrogen). Cells were transfected with plasmids and siRNAs using Lipofectamine 2000 according to the manufacturer’s instructions (Invitrogen). For production of stable cell lines, cells were selected with media containing either 15 μg/ml blasticidin (for vector pcDNA6.V5-His) or 500 μg/ml geneticin sulphate (G418) (for vector pEGFPC1) for 4 weeks (Santa Cruz Biotechnology). Transiently transfected cells were analysed 16–24 h post-transfection and siRNA treated cells 72 h post-transfection. Rat cortical neurons were prepared and transfected with Lipofectamine 2000 as previously described [81].

Induced pluripotent stem (iPS) cells from a familial Parkinson’s disease patient carrying gene triplication of *SNCA* encoding α-synuclein (α-synuclein triplication; AST cells) and an unaffected first-degree relative control (normal α-synuclein; NAS cells) were maintained and differentiated into dopaminergic cells as described [21, 89]. 54–60% of the cells were neuronal based upon immunostaining for markers. Two different disease AST clones and two different control NAS clones were used in the studies and pooled data shown. For analyses, iPS cell-derived neurons were grown on 35 mm IBIDI dishes (BD Biosciences) as described [21].

### SDS-PAGE and immunoblotting

Cells were harvested for SDS-PAGE and immunoblotting by scraping into SDS-PAGE sample buffer containing 2% SDS, 100 mM dithiothreitol, 10% glycerol, 0.1% bromophenol blue and protease inhibitors (Complete Roche) in 50 mM Tris–HCl pH 6.8 and heating to 100 °C for 5 min. Other samples were prepared by addition of SDS-PAGE sample buffer and heating to 100 °C for 5 min. Samples were separated on 8–15% (w/v) acrylamide gels and transferred to Protran nitrocellulose membranes (Schleicher and Schuell) using a Mini-PROTEAN 3 gel electrophoresis system and Transblot system (BioRad). The immunoblots were then blocked by incubation in 5% (w/v) dried milk/0.1% (w/v) Tween-20 in Tris buffered saline (TBS) pH 7.5 for 1 h and then probed with primary antibodies diluted in blocking solution for 16 h at 4 °C. Following washing in blocking solution, they were then incubated with horseradish peroxidase-conjugated goat anti-mouse, anti-rabbit or anti-rat Igs (GE Healthcare). Immunoblots were developed using an enhanced chemiluminescence Luminata Forte Western HRP substrate system according to the manufacturer’s instructions (Millipore). Signals on immunoblots were quantified using ImageJ after scanning with an Epson Precision V700 Photo scanner essentially as described by us in previous studies [78].

### Immunoprecipitation and glutathione S-transferase (GST) pull-down assays

For immunoprecipitations, cells were harvested in ice-cold lysis buffer containing 1% Triton X-100 and Complete protease and PhosStop inhibitor cocktail tablets (Roche) in phosphate-buffered saline (PBS) and then lysed for 30 min on ice. The lysates were then cleared by centrifugation at 100,000×*g* for 30 min at 4 °C and 500 μg protein incubated with primary antibodies for 16 h at 4 °C. Antibodies were captured with protein A or protein G Sepharose beads (Sigma) for 2 h at 4 °C depending upon Ig type and the beads washed in ice cold lysis buffer. Samples were then heated to 100 °C in SDS-PAGE sample buffer and analysed by SDS-PAGE and immunoblotting. For brain samples, rat brains were homogenized in ice-cold lysis buffer and treated as above.

Recombinant GST, GST-VAPB fragments and α-synuclein were produced in *E. coli* BL21 (DE3). GST tagged proteins were purified on glutathione-Sepharose beads according to the manufacturer’s instructions (GE Healthcare) and as described by us previously [78]. α-Synuclein was purified as described [73]. For pull-down assays, GST or GST-VAPB proteins were incubated overnight with 50 μg purified recombinant α-synuclein in ice-cold lysis buffer containing PBS with 1% Triton X-100 and Complete protease and PhosStop inhibitor cocktail tablets (Roche). GST-fusion protein complexes were pulled down via the glutathione-Sepharose beads and washed three times with ice-cold lysis buffer prior to analyses by SDS-PAGE and immunoblotting. For cellular GST pull-down assays, baits were incubated with cell lysates and captured essentially as described above. HEK293 cells were used for immunoprecipitation and GST pull down assays since they transfect with high efficiency and so produce robust readouts that are particularly suitable for such biochemical studies.

### Biochemical fractionation

ER, MAM and mitochondria were prepared from adult Sprague–Dawley rat brains essentially as described [87]. Briefly, fresh rat brains were washed in ice-cold isolation buffer containing 225 mM mannitol, 75 mM sucrose, 0.5% BSA and 0.5 mM EGTA in 30 mM Tris–HCl buffer pH 7.4. All procedures were then performed on ice. Three brains were chopped into fine pieces and washed three times in isolation buffer and then prepared as a 10% w/v mixture in isolation buffer. Tissues were homogenized using a Dounce homogenizer (20 strokes by hand using the tightest pestle) and the homogenate centrifuged twice at 740×*g* for 5 min to remove nuclei and unbroken cells. The crude mitochondrial fraction was then pelleted by centrifugation at 9000×*g* for 10 min and the remaining supernatants were centrifuged at 100,000×*g* for 30 min to pellet ER/microsomes. To isolate MAM and pure mitochondria, the crude mitochondrial pellet was resuspended in isolation buffer and layered on top of a self-forming 30% Percoll gradient (225 mM mannitol, 1 mM EGTA, 0.05% BSA, 30% Percoll, 25 mM Na-HEPES pH 7.4). After centrifugation at 95,000×*g* for 30 min the mitochondria were recovered at the bottom of the gradient and MAM retrieved above. To remove residual Percoll, the mitochondrial fraction was diluted in mitochondria resuspending buffer (5 mM HEPES pH 7.4 containing 250 mM mannitol and 0.5 mM EGTA) and the mitochondria washed twice by centrifugation at 6300×*g* for 10 min. The MAM band was diluted with isolation buffer and centrifuged once at 6300×*g* for 10 min to remove any residual contaminating mitochondria. MAM was then pelleted from the resulting supernatant by centrifugation at 100,000×*g* for 1 h. All final organelle pellets were resuspended and lysed in in RIPA buffer containing 50 mM Tris–HCl pH 6.8 containing 150 mM NaCl, 1 mM EDTA, 1 mM EGTA, 1% Triton X-100, 0.5% sodium deoxycholate, 0.1% SDS with Complete protease inhibitor (Roche). Protein concentrations were determined using a Bradford assay (Bio-Rad Laboratories).

### Electron microscopy

Cells were fixed with 2.5% glutaraldehyde in 0.1 M sodium cacodylate buffer pH 7.2 for 3 h at 20 °C and then harvested by a gentle scraping with a plastic scaper. The cells were pelleted by centrifugation at 800 g (av) for 10 min, washed in 0.1 M sodium cacodylate buffer and post-fixed for 1 h in 2% osmium tetroxide and 1.5% ferricyanide in 0.1 M sodium cacodylate buffer. The cells were then stained for 1 h with 1% uranyl acetate in water before dehydration and embedding in epoxy resin (TAAB). 150 nm semi-fine sections were cut on a Reichert Ultra cut E ultramicrotome and stained for 6 min in 0.16% lead citrate in 0.1 M NaOH followed by 3 washes in distilled water. Samples were viewed on a Tecnai 12 electron microscope at 4800× magnification. Digital images were acquired and the circumference of each mitochondria and the proportions of the mitochondrial surface closely associated (<30 nm) with ER were calculated. Cells were randomly selected for analyses without prior knowledge of transfected plasmid or siRNA. All clearly identified mitochondria in the samples were scored and the numbers of mitochondria and ER profiles were quantified from lower magnification images. Image analyses were performed using ImageJ.

### Light microscopy

For super resolution structured illumination microscopy, cells plated on glass coverslips were fixed with 3% paraformaldehyde and 0.1% glutaraldehyde in PBS for 10 min at 20 °C. After washing with PBS, samples were quenched by incubation with 50 mM NaBH_4_ in PBS for 7 min, washed in PBS and then permeabilized and blocked for 30 min in PBS containing 0.2% Triton X-100 and 3% bovine serum albumin (BSA). Samples were then incubated with primary antibodies diluted in blocking solution, washed with PBS and incubated with goat anti-rabbit or anti-mouse secondary Igs conjugated to AlexaFluor 546 or AlexaFluor 633 (Invitrogen). Following final washings in PBS, the samples were mounted in Mowiol-DABCO mounting medium containing 10% (w/v) Mowiol 4-88 (Calbiochem), 25% (w/v) glycerol and 2.5% (w/v) DABCO (1,4-diazobicyclo[2.2.2]octane) in 100 mM Tris–HCl pH 8.5. Samples were analysed on a Nikon Eclipse Ti-E inverted microscope equipped with a Nikon N-SIM Super Resolution System. Images were captured using a CFI Plan Apo IR SR 60× water immersion objective and then reconstructed using Nikon Imaging Software Elements AR with N-SIM module. ER–mitochondria interactions were quantified by Mander’s coefficient using ImageJ software. Co-localized signals were displayed using the ImageJ 1.44p RG2B co-localization plugin to determine co-localized pixels.

For confocal and wide-field microscopy, cells were fixed in 4% paraformaldehyde in PBS, immunostained essentially as described [28] and analysed using a Leica TCS-SP5 confocal with x63HCX PL APO lambda blue CS 1.4 objective or a Leica DM5000 fluorescence microscope equipped with a 40×/0.75NA HCX-PL-FLUOTAR lens. Mitochondria and ER morphologies and cellular distributions were determined using ImageJ with the Analyse Particle function and Mitochondrial Morphology [17] and AnalysedSkeleton [3] Plugins.

Proximity ligation assays to quantify VAPB-PTPIP51 and α-synuclein-VAPB interactions were performed essentially as described previously using Duolink reagents (Sigma-Aldrich) [20, 28, 78]. Cells were fixed in 4% paraformaldehyde in PBS and probed with primary antibodies. Signals were developed using the Duolink In Situ Orange kit. Cells were counterstained with 4′,6-diamidino-2-phenylindole (DAPI) to show nuclei and/or further immunostained for NFH or MAP2. Proximity ligation assay signals were quantified using the Particle Analysis function of ImageJ.

### Ca^2+^ measurements

Ca^2+^ measurements were performed essentially as described previously [20, 78, 79]. SH-SY5Y cells were loaded with 2 μM Fluo4-AM and/or Rhod2-AM dye (Invitrogen) in external solution (145 mM NaCl, 2 mM KCl, 5 mM NaHCO_3_, 1 mM MgCl_2_, 2.5 mM CaCl_2_, 10 mM glucose, 10 mM Na-HEPES, pH 7.25) containing 0.02% Pluronic-F27 (Invitrogen) for 15 min at 37 °C, followed by washing in external solution for 15 min at 37 °C. Fluo4 and Rhod2 fluorescence were timelapse recorded (1 s intervals) at 37 °C using either an Axiovert S100 microscope (Zeiss) driven by MetaMorph (Molecular Dynamics) and equipped with GFP (Fluo4) and DsRed (Rhod2) filtersets (Chroma Technology), a 40× Plan-Neofluar 1.3NA objective (Zeiss), and a Photometrics Cascade-II 512B36 EMCCD camera or a Nikon Ti-E microscope using a CFI Plan Apo VC 20× objective and Nikon Andor Neo sCMOD high-resolution camera and appropriate filter sets. The cells were kept under constant perfusion with external solution (0.5 ml/min). Inositol 1,4,5-trisphosphate (IP3) receptor-mediated Ca^2+^ release from ER stores was triggered by application of 100 μM oxotremorine-M for 2 min. Ca^2+^ levels were calculated as relative Rhod2 or Fluo4 fluorescence compared to baseline fluorescence (*F*/*F*_0_) at the start of the measurement. Oxotremorine-M was from Santa Cruz Biotechnology and was dissolved in water.

### ATP measurements

ATP levels in cultured cells were measured using a ViaLight ATP kit (Lonza) according to the manufacturer’s instructions; luminescence signals were obtained with a FluoSTAR luminometer (BMG Labtech). To determine ATP levels generated by oxidative phosphorylation in mitochondria, cells were first treated with 100 μM iodoacetate (Sigma) for 2 h to inhibit ATP produced by glycolysis. ATP levels were also determined using a FRET based plasmid reporter (Adenosine 5′-Triphosphate indicator based on Epsilon subunit for Analytical Measurements; ATeam reporter) [42]. To do so, cells were transfected with AT1.03 cytosolic ATeam reporter and then imaged in Hanks Balanced Salt Solution (HBSS) without phenol red at 37 °C by timelapse microscopy (12 s intervals) on a Zeiss Axiovert S100 microscope equipped with a 40×/1.3NA Plan-Neofluar objective and a Photometrics Cascade-II 512B EMCCD driven by MetaMorph (Molecular Dynamics). FRET filtersets (ECFP excitation filter ET 430/24×; ECFP emission filter 470/24 m; EYFP emission filter ET545/40m) were from Chroma Technology. 1 mM KCN in HBSS was applied using a peristaltic pump (0.5 ml/min). YFP/CFP ratios prior to and after KCN treatment were measured as described and used to calculate relative ATP levels in the different samples which were displayed as bar charts [42].

### Statistical analyses

All experiments were repeated at least three times. Statistical analyses were performed with Prism 5.0 (GraphPad Software).

## Results

### Wild-type and familial Parkinson’s disease mutant α-synuclein disrupt ER–mitochondria associations and the VAPB-PTPIP51 interaction

**Fig. 1:**
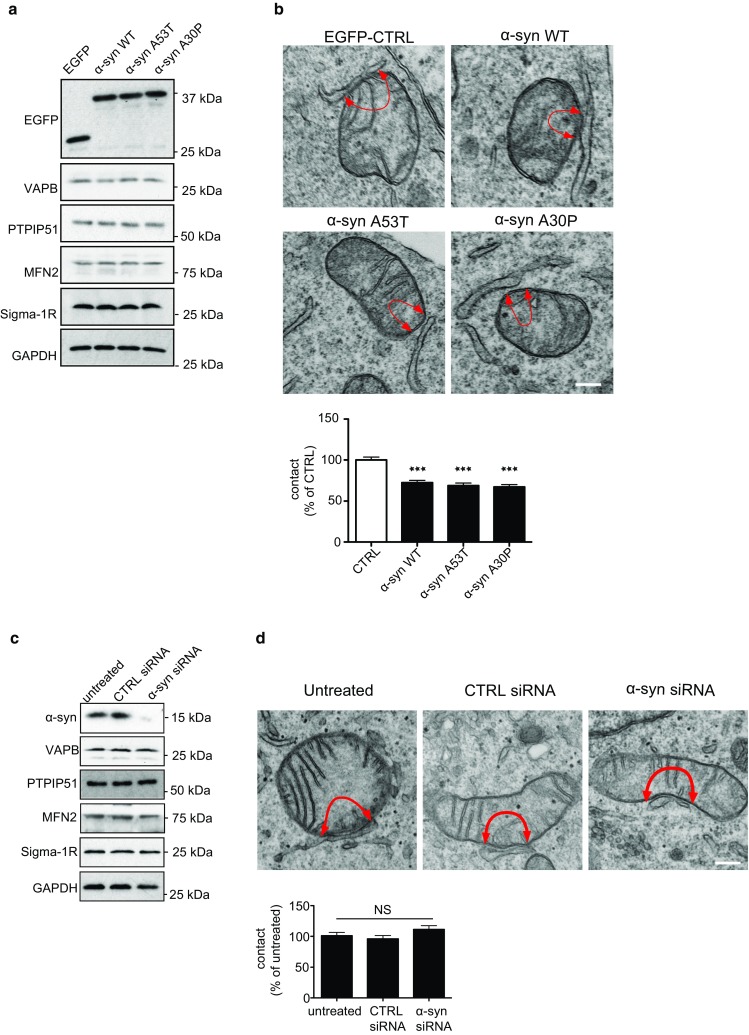
Expression of wild-type and familial Parkinson’s disease mutant α-synuclein reduce ER–mitochondria associations in SH-SY5Y cells. **a** Expression of α-synuclein does not alter expression of VAPB, PTPIP51 mitofusin-2 (MFN2) or the Sigma-1 receptor in stably transfected SH-SY5Y cells. Immunoblots of SH-SY5Y cells stably transfected with EGFP as a control, EGFP-α-synuclein, EGFP-α-synucleinA53T or EGFP-α-synucleinA30P and probed on immunoblots as indicated; GAPDH is shown as a loading control. Molecular masses in kD are shown on the right. **b** Representative electron micrographs of ER–mitochondria associations in SH-SY5Y cells expressing control EGFP vector (CTRL), EGFP-α-synuclein, EGFP-α-synucleinA53T or EGFP-α-synucleinA30P; *arrowheads* with loops show regions of association. *Scale bar* is 200 nm. *Bar chart* shows % of the mitochondrial surface closely apposed to ER in the different samples. Data were analysed by one-way ANOVA followed by Tukey’s multiple comparison test. *N* = 30–35 cells and 107–155 mitochondria; *error bars* are SEM; ***p < 0.001. **c**, **d** siRNA loss of α-synuclein does not alter expression of VAPB, PTPIP51, mitofusin-2 (MFN2) or the Sigma-1 receptor, or affect ER–mitochondria associations in SH-SY5Y cells. **c** Immunoblots of cells either untreated or treated with control (CTRL) or α-synuclein siRNAs; GAPDH is shown as a loading control. Molecular masses in kD are shown on the right. **d** Representative electron micrographs of ER–mitochondria associations in untreated, control (CTRL) and α-synuclein siRNA treated cells. *Arrowheads* with loops show regions of association. *Bar chart* shows % of the mitochondrial surface closely apposed to ER in the different samples. Data were analysed by one-way ANOVA followed by Tukey’s multiple comparison test. *N* = 30–32 cells and 66–99 mitochondria. *Error bars* are SEM; *NS* not significant. *Scale bar* is 200 nm

To determine the effects of α-synuclein on ER–mitochondria associations, we quantified ER–mitochondria contacts in polyclonal populations of SH-SY5Y cells stably expressing either enhanced green fluorescent protein (EGFP) control vector, EGFP-α-synuclein or familial Parkinson’s disease mutant EGFP-α-synucleinA53T or EGFP-α-synucleinA30P. Numerous studies have utilized α-synuclein tagged with EGFP in this way (e.g. [35, 61, 64, 72, 77]). Probing of immunoblots with an EGFP antibody revealed that they expressed similar levels of exogenous protein and that expression of α-synuclein did not affect expression of the ER–mitochondria tethering proteins VAPB and PTPIP51, of mitofusin-2 which has been proposed as a further ER–mitochondria tether, or of the Sigma-1 receptor which has also been linked to ER–mitochondria tethering [5, 19, 29, 86] (Fig. 1a). Likewise, no changes in the levels of TOM20 or PDI were detected (Supplemental Fig. 1a). The absence of changes in the levels of PTPIP51, TOM20 and mitofusin-2 (mitochondrial proteins) or of VAPB and PDI (ER proteins) suggest that there are no overall changes in mitochondrial or ER masses in the different transfected cells. We also quantified the numbers of mitochondria and ER profiles, and mitochondrial circumferences in the EM and detected no differences between the different transfected cells (data obtained from 30 to 35 cells; analysed by one-way ANOVA). However, confocal and structured illumination microscopy (SIM) revealed that mitochondria in EGFP-α-synuclein and EGFP-α-synucleinA53T cells had increased circularity (rounding up) (EGFP-α-synucleinA30P displayed a trend for increased circularity) and that mitochondria in wild-type and mutant EGFP-α-synuclein cells had reduced cytosolic distributions (increased clustering) (Supplemental Fig. 1b, c). We detected no changes in ER cytosolic distributions, the numbers of ER branch points or branch length (i.e. overall ER morphology) in wild-type or mutant EGFP-α-synuclein cells compared to control (Supplemental Fig. 1d, e, f).

ER–mitochondria associations were, therefore, quantified by determining the proportion of the mitochondrial surface that was closely apposed (less than 30 nm) to ER following analyses by EM. This and similar EM approaches have been used previously [15, 16, 23, 24, 26, 52, 78, 85]. Compared to control cells, expression of wild-type and mutant α-synuclein all led to significant reductions in ER–mitochondria associations (Fig. 1b).

We also enquired whether loss of α-synuclein influenced ER–mitochondria associations. To do so, we quantified ER–mitochondria associations by EM in untreated SH-SY5Y cells and in cells treated with control or α-synuclein siRNAs. Probing of samples on immunoblots revealed that the α-synuclein siRNAs produced an approximate 90% knockdown of α-synuclein but this did not affect expression of VAPB, PTPIP51, mitofusin-2 or the Sigma-1 receptor (Fig. 1c). However, compared to untreated or control siRNA treated cells, the EM studies revealed no change in ER–mitochondria associations in the α-synuclein knockdown cells (Fig. 1d).

**Fig. 2:**
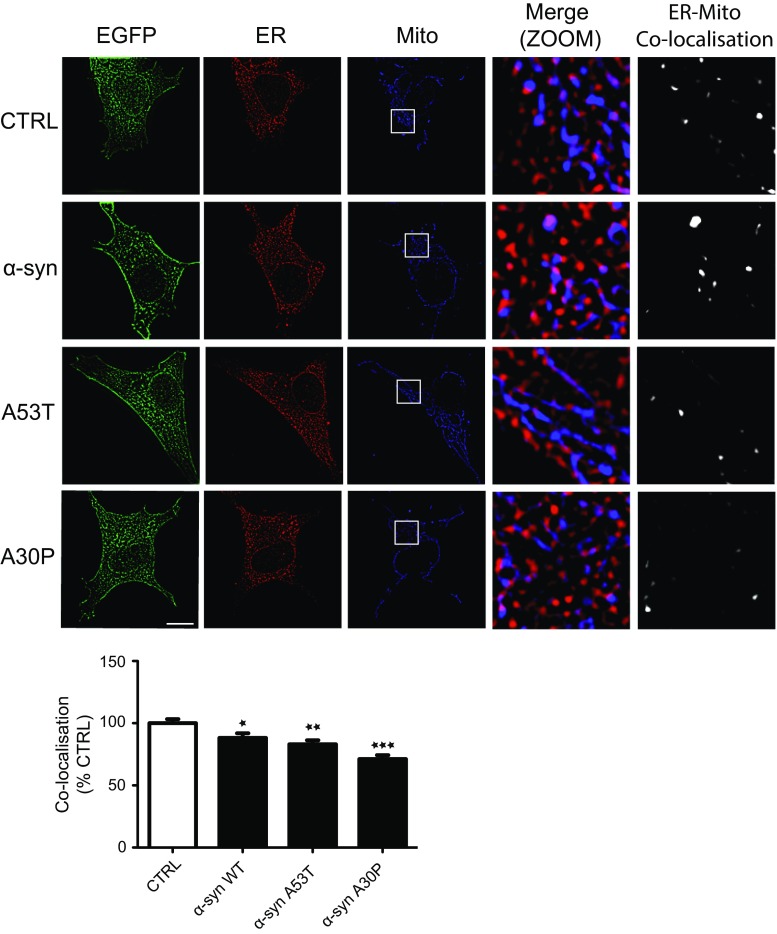
Super resolution SIM reveals reduced ER–mitochondria associations and VAPB–PTPIP51 interactions in SH-SY5Y cells expressing wild-type or mutant α-synuclein. SH-SY5Y cells were transfected with either EGFP control vector (CTRL), EGFP-α-synuclein (α-syn), EGFP-α-synucleinA53T (A53T) or EGFP-α-synucleinA30P (A30P) and immunostained for PDI and TOM20 to label ER and mitochondria (Mito), respectively; α-synuclein were detected via their EGFP tags. Merge (ZOOM) show zoomed images of boxed regions and co-localisation shows co-localised pixels. *Scale bar* is 15 μm. *Bar chart* shows ER–mitochondria co-localisation (Manders coefficient) normalized to control in the different samples. Data were analysed by one-way ANOVA with Tukey’s post hoc test. 30 cells were analysed per condition from 3 independent experiments; *error bars* are SEM, *p < 0.05, **p < 0.01, ***p < 0.001

To complement the EM studies, we monitored the effects of α-synuclein expression on ER–mitochondria associations using super resolution structured illumination microscopy (SIM) [39]. For these studies, SH-SY5Y cells were transfected with EGFP control vector, EGFP-α-synuclein, EGFP-α-synucleinA53T or EGFP-α-synucleinA30P and then immunostained for PDI and TOM20 to label ER and mitochondria, respectively. Co-localisation analyses of PDI and TOM20 revealed that wild-type and mutant α-synuclein again disrupted ER–mitochondria associations (Fig. 2). Further analyses of these images revealed that a proportion of α-synuclein localized with the co-localized PDI/TOM20 signals (i.e. some α-synuclein is present at ER–mitochondria contact sites) (Supplemental Fig. 2).

**Fig. 3:**
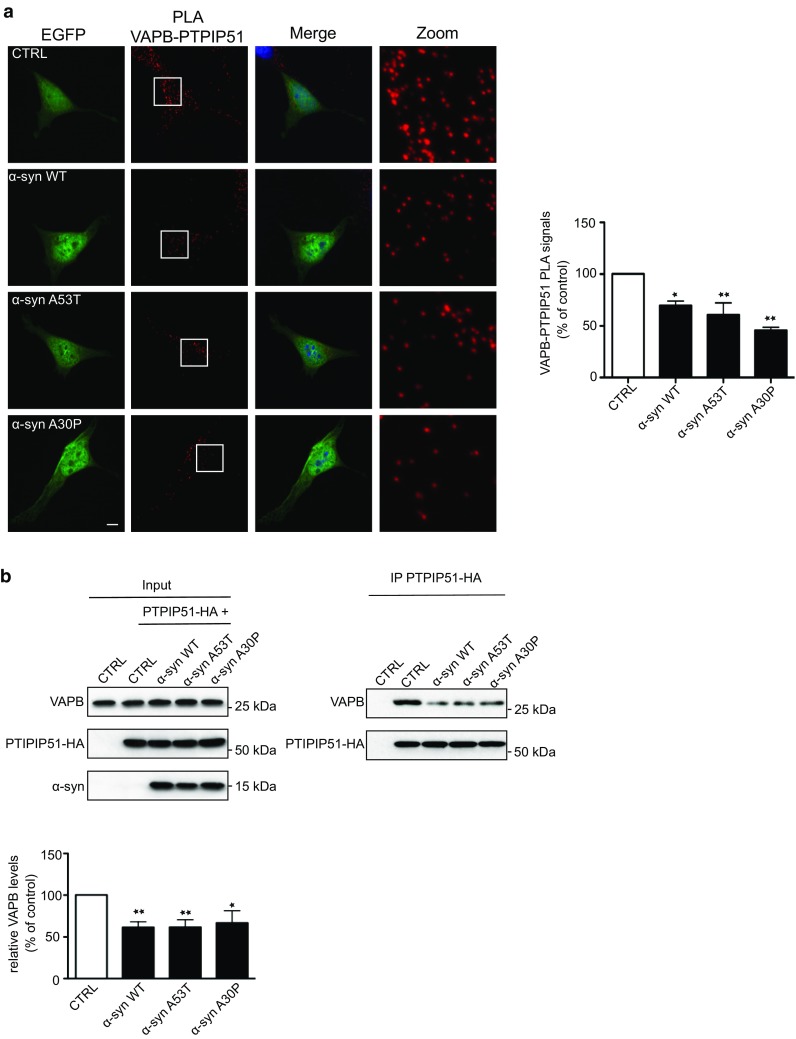
Proximity ligation assays and immunoprecipitation experiments reveal reduced ER–mitochondria associations and VAPB–PTPIP51 interactions in SH-SY5Y cells expressing wild-type or mutant α-synuclein. **a** VAPB-PTPIP51 proximity ligation assays in SH-SY5Y cells transfected with EGFP-control vector (CTRL), EGFP-wild-type α-synuclein (α-syn WT), α-synucleinA53T (α-syn A53T) or α-synucleinA30P (α-syn A30P). Representative maximum intensity projections of serial confocal optical sections of cells transfected with the different plasmids are shown. Zoom shows high magnification proximity ligation assay signals in boxed regions. Proximity ligation assays were performed using rabbit VAPB and rat PTPIP51 antibodies. *Bar chart* shows proximity ligation assay signals (normalised to control) in the different samples. Data were analysed by one-way ANOVA and Tukey’s post hoc test. *N* = 176–255 cells from 3 different experiments; *error bars* are SEM, *p < 0.05, **p < 0.01. *Scale bar* is 5 μm. **b** VAPB-PTPIP51 immunoprecipitation assays in SH-SY5Y cells transfected with CAT control vector (CTRL), CTRL + PTPIP51-HA or PTPIP51-HA + either α-synuclein, α-synucleinA53T, α-synucleinA30P. PTPIP51 was immunoprecipitated via the HA tag and endogenous bound VAPB detected by immunoblotting. No signals for either VAPB or PTPIP51 were detected in immunoprecipitations from CTRL transfected cells which demonstrates the specificity of the assays. Both inputs and immunoprecipitations (IP) are shown. Extended exposures of the blots revealed the presence on endogenous α-synuclein in control cells. Molecular masses in kD are shown on the right. *Bar chart* shows relative levels of VAPB bound to PTPIP51 in the immunoprecipitations following quantification of signals from immunoblots. VAPB signals were normalized to immunoprecipitated PTPIP51-HA signals. Data were analysed by one-way ANOVA and Tukey’s post hoc test. *N* = 5; *error bars* are SEM, *p < 0.05, **p < 0.01

We also used in situ proximity ligation assays [75] to monitor the effects of α-synuclein on ER–mitochondria associations. Proximity ligation assays have a resolution similar to that detected by resonance energy transfer between fluorophores (i.e. approximately 10 nm) [75] and so are suitable for quantifying ER–mitochondria associations. For these assays, we used VAPB and PTPIP51 primary antibodies as markers for ER and mitochondria, respectively, since these proteins interact directly to tether ER with mitochondria [20, 78, 79]. Indeed, proximity ligation assays have already been used to quantify ER–mitochondria associations and the VAPB-PTPIP51 interaction [5, 20, 36]. We have previously demonstrated the specificity of these VAPB-PTPIP51 proximity ligation assays via control experiments involving the absence of each or both primary antibodies [20, 79]. Transfection of SH-SY5Y cells with EGFP-α-synuclein, EGFP-α-synucleinA53T or EGFP-α-synucleinA30P all produced significant reductions in ER mitochondria associations in these assays (Fig. 3a).

Since we detected no α-synuclein-induced changes in expression of VAPB or PTPIP51 (Fig. 1a, c), these proximity ligation assays not only confirm that α-synuclein reduces ER–mitochondria associations, but also show that this reduction involves breaking of the VAPB-PTPIP51 tethers. To test this further, we performed immunoprecipitation assays to monitor the effect of wild-type and mutant α-synuclein on binding of VAPB to PTPIP51. To do so, we co-transfected cells with hemagglutinin (HA)-tagged PTPIP51 and either control vector, α-synuclein, α-synucleinA53T or α-synucleinA30P and monitored the amounts of VAPB bound to immunoprecipitated PTPIP51-HA by immunoblotting of the samples. Consistent with the proximity ligation assays, both wild-type and mutant α-synuclein decreased the amounts of endogenous VAPB bound to immunoprecipitated PTPIP51-HA in these assays (Fig. 3b).

### The VAPB-PTPIP51 interaction is disrupted in dopaminergic neurons derived from iPS cells that carry a familial Parkinson’s disease α-synuclein gene triplication

**Fig. 4:**
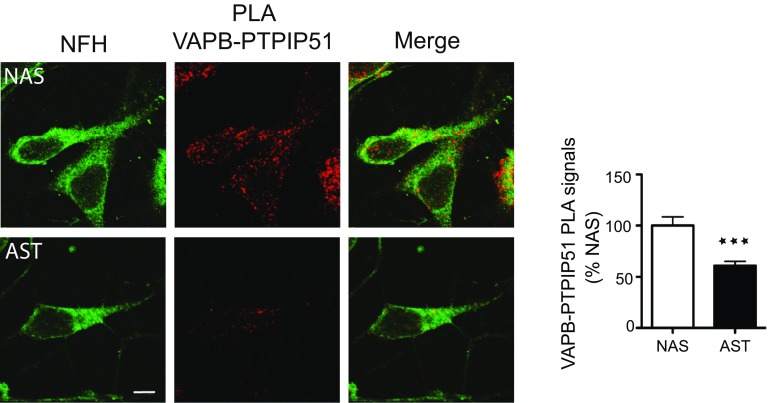
ER–mitochondria associations and the VAPB–PTPIP51 interaction are reduced in dopaminergic neurons derived from iPS cells harbouring triplication of the α-synuclein gene. Representative images of VAPB-PTPIP51 proximity ligation assays in NAS control and AST dopaminergic neurons; cells were also stained for neurofilament heavy chain (NFH) to confirm neuronal phenotype. *Scale bar* is 10 μm. *Bar chart* shows quantification of proximity ligation assay signals in the samples. Data were analysed by Student’s *t* test. *N* = 74–90 cells from 3 different experiments; *error bars* are SEM, ***p < 0.001

To determine whether α-synuclein also disrupts the VAPB–PTPIP51 interaction in Parkinson’s disease patient material, we utilized proximity ligation assays to probe ER–mitochondria associations and the VAPB-PTPIP51 interaction in iPS cell-derived dopaminergic neurons from a *SNCA* triplication patient (α-synuclein triplication; AST) and a non-disease first degree relative control (normal α-synuclein; NAS) [21]. *SNCA* triplication causes autosomal dominant familial Parkinson's disease and neurons carrying this mutation have higher levels of α-synuclein compared to controls [21, 41, 74]. Compared to controls, the VAPB-PTPIP51 proximity ligation assay signals were significantly reduced in the *SNCA* triplication neurons (Fig. 4). Thus, expression of both wild-type and mutant α-synuclein reduces ER–mitochondria associations and the VAPB–PTPIP51 interaction in a number of different assays and this includes in dopaminergic neurons derived from familial Parkinson’s disease patients carrying *SNCA* gene triplication.

### Wild-type and mutant α-synuclein disrupt IP3 receptor-mediated delivery of Ca^2+^ from ER stores to mitochondria

**Fig. 5:**
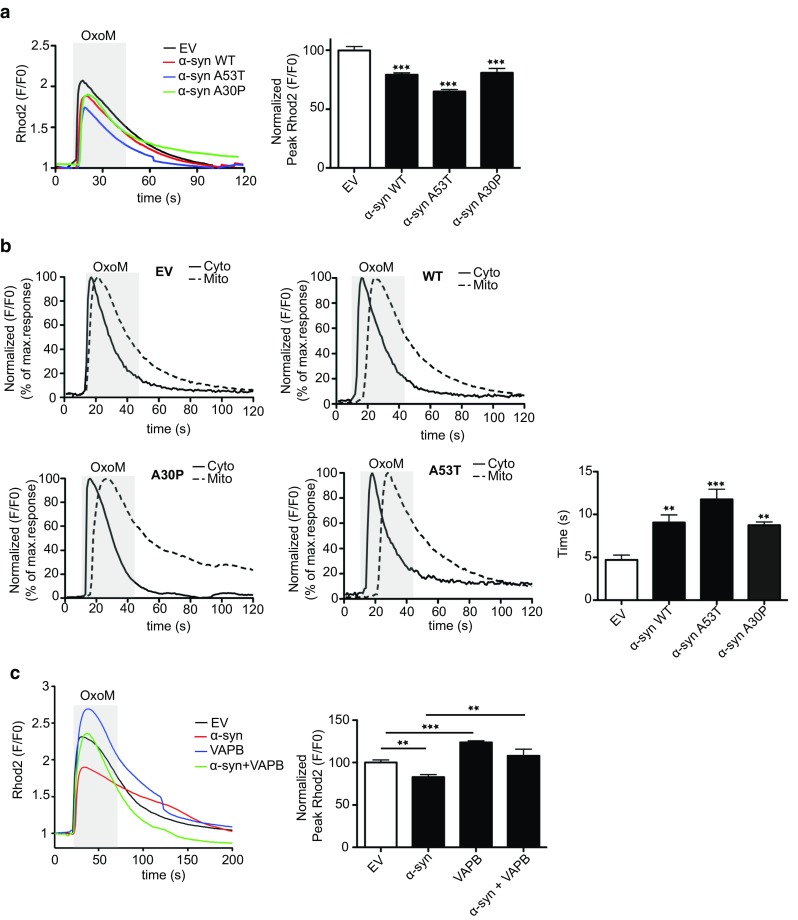
ER–mitochondria Ca^2+^ exchange is disrupted in SH-SY5Y cells stably expressing α-synuclein and overexpression of VAPB to increase ER–mitochondria contacts rescues disrupted Ca^2+^ exchange. **a**, **b** Reduced mitochondrial Ca^2+^ uptake and delayed ER–mitochondrial Ca^2+^ exchange following IP3 receptor-mediated release from ER-stores in α-synuclein, α-synucleinA53T and α-synucleinA30P expressing cells. IP3 receptor-mediated Ca^2+^ release was triggered with oxotremorine-M (OxoM) and mitochondrial and cytosolic Ca^2+^ levels detected by Rhod2 and Fluo4 fluorescence, respectively. **a** Representative Rhod2 fluorescence traces are shown with normalized peak values (*F*/*F*_0_) in the *bar chart*. Data were analysed by one-way ANOVA and Tukey’s post hoc test. *N* = 29–44 cells from 3 different experiments; *error bars* are SEM, ***p < 0.001. **b** Delayed mitochondrial Ca^2+^ uptake following IP3 receptor-mediated release from ER-stores in α-synuclein, α-synucleinA53T and α-synucleinA30P expressing cells. Representative Fluo4 (cytosolic; Cyto) and Rhod2 (mitochondria; mito) fluorescence traces are shown. *Bar chart* shows the time-lag between peak cytosolic and mitochondrial Ca^2+^ signals. Data were analysed by one-way ANOVA and Tukey’s post hoc test. *N* = 85–115 cells from 4 independent experiments; *error bars* are SEM, **p < 0.01 and ***p < 0.001. **c** Expression of VAPB to increase ER–mitochondria contacts increases mitochondrial Ca^2+^ levels following IP3 receptor-mediated release and rescues defective Ca^2+^ uptake induced by α-synuclein. Representative Rhod2 fluorescence traces are shown with normalized peak values (*F*/*F*_0_) in the *bar chart*. Data were analysed by one-way ANOVA and Tukey’s post hoc test. *N* = 16–72 cells from three different experiments; *error bars* are SEM, **p < 0.01, ***p < 0.001

A primary function of ER–mitochondria associations is to facilitate delivery of Ca^2+^ to mitochondria from ER stores. This delivery is mediated via IP3 receptors located in MAM [16, 19, 46, 48, 63, 70, 82]. Thus, disruption to the VAPB–PTPIP51 interaction via siRNA loss of VAPB or PTPIP51 to loosen ER–mitochondria associations perturbs this delivery [20, 78, 79]. Since expression of wild-type and mutant α-synuclein reduces both ER–mitochondria associations and the VAPB–PTPIP51 interaction, we, therefore, monitored the effect of α-synuclein on mitochondrial Ca^2+^ uptake following its release from ER stores. For these experiments, we used SH-SY5Y cells stably expressing control empty vector, α-synuclein, α-synucleinA53T or α-synucleinA30P and triggered physiological IP3 receptor-mediated Ca^2+^ release from ER by application of the M3 muscarinic acetylcholine receptor agonist oxotremorine-M. This approach has been utilized previously [20, 78, 79]. In agreement with previous studies [20, 78, 79], oxotremorine-M induced a time-dependent increase in mitochondrial Ca^2+^ levels (Fig. 5a). However, compared to controls, the peak values were lower in cells expressing wild-type or mutant α-synuclein (Fig. 5a). This finding suggests that α-synuclein perturbs Ca^2+^ delivery to mitochondria. To test this further, we monitored cytosolic and mitochondrial Ca^2+^ levels following oxotremorine-M mediated Ca^2+^ release from ER. Compared to controls, the time-lags between cytosolic and mitochondrial peak values were longer in cells expressing wild-type and mutant α-synuclein (Fig. 5b). Finally, we enquired whether the reduced Ca^2+^ uptake by mitochondria in α-synuclein expressing cells following IP3 receptor-mediated release could be rescued by transfection of VAPB to restore ER–mitochondria associations; VAPB overexpression increases ER–mitochondria contacts [78]. In agreement with previous studies [28], transfection of VAPB increased mitochondrial Ca^2+^ uptake (Fig. 5c). Moreover, VAPB overexpression completely abrogated the effect of α-synuclein on mitochondrial Ca^2+^ uptake (Fig. 5c). Thus, α-synuclein induced breaking of the VAPB-PTPIP51 tethers and loosening of ER–mitochondria associations are accompanied by disruption to delivery of Ca^2+^ to mitochondria from ER stores.

### Wild-type and mutant α-synuclein inhibit mitochondrial ATP production

**Fig. 6:**
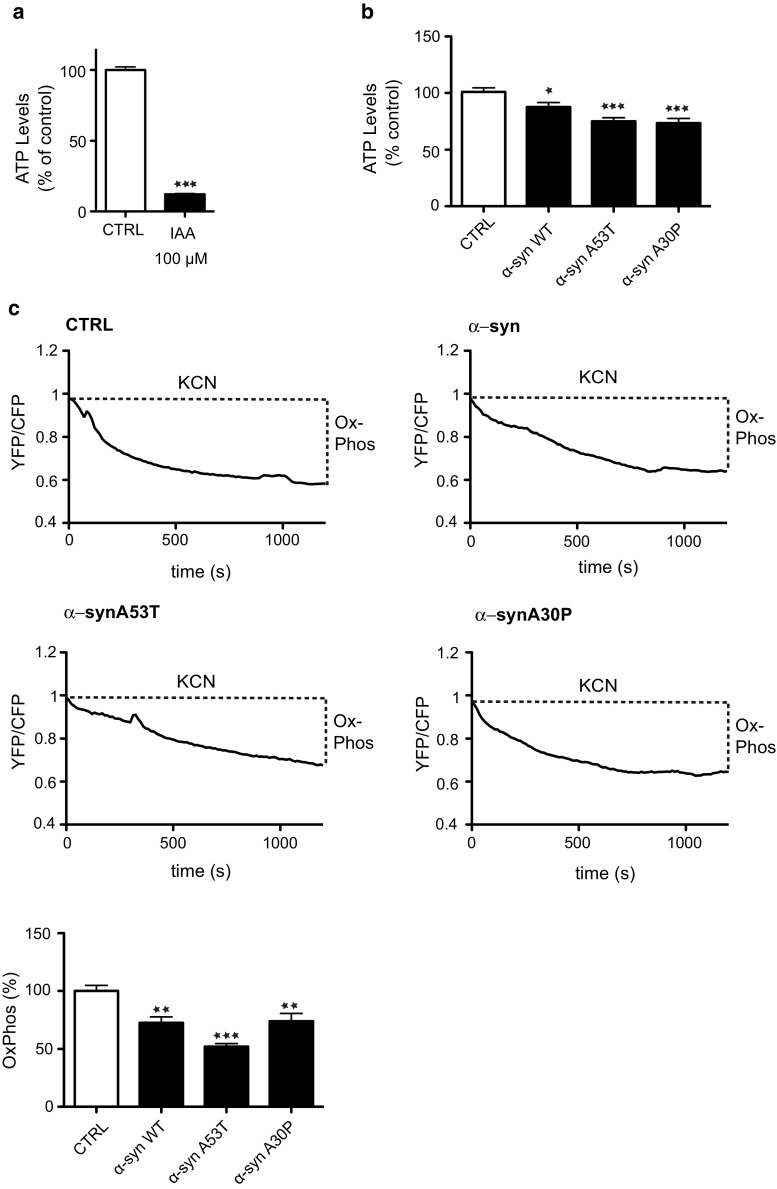
Reduced mitochondrial ATP production in SH-SY5Y cells stably expressing α-synuclein, α-synucleinA53T or α-synucleinA30P. **a** ATP levels in SH-SY5Y cells treated with either vehicle (H_2_O) or 100 μM iodoacetate (IAA) for 2 h to inhibit glycolysis and measured using ViaLight assay. Data were analysed by Student’s *t* test. *N* = 3; *error bars* are SEM, ***p < 0.001. **b** ATP levels in stably transfected SH-SY5Y cells expressing control empty vector, wild-type α-synuclein, α-synucleinA53T or α-synucleinA30P and treated with 100 μM iodoacetate for 2 h. Data were analysed by one-way ANOVA and Tukey’s post hoc test. *N* = 4; *error bars* are SEM, *p < 0.05 and ***p < 0.001. **c** ATP levels were measured in SH-SY5Y cells transfected with the ATP indicator AT1.03. Cells were imaged in time-lapse and treated with KCN to inhibit oxidative phosphorylation. Representative traces of YFP/CFP ratios are shown for the different samples. The fall in YFP/CFP ratios correlates with ATP produced by oxidative phosphorylation. *Bar chart* shows relative ATP levels produced by oxidative phosphorylation (OxPhos) in the different samples. Data were analysed by one-way ANOVA and Tukey’s post hoc test. *N* = 15–27 cells from 3 experiments, *error bars* are SEM; **p < 0.01, ***p < 0.001

Ca^2+^ is required by mitochondria for generating ATP via the tricarboxylic acid cycle [30] and so the reduced mitochondrial Ca^2+^ levels seen in both wild-type and mutant α-synuclein expressing cells predict that α-synuclein impairs mitochondrial ATP production. We, therefore, monitored mitochondrial ATP production in the stably expressing α-synuclein SH-SY5Y cells. To do so we used a bioluminescent assay that measures total cellular ATP levels (generated by both glycolysis and oxidative phosphorylation) and inhibited glycolytic ATP production with iodoacetic acid. This method has been used by others to measure ATP generated by oxidative phosphorylation in mitochondria [4]. To first confirm the effect of iodoacetic acid, we measured ATP production in control SH-SY5Y cells treated with either vehicle or iodoacetic acid; as predicted, iodoacetic acid markedly reduced ATP levels (Fig. 6a). We then measured ATP levels in the different transfected cells following treatment with iodoacetic acid. Compared to control cells, the levels of ATP generated by oxidative phosphorylation were significantly reduced in cells expressing α-synuclein, α-synucleinA53T and α-synucleinA30P (Fig. 6b).

**Fig. 7:**
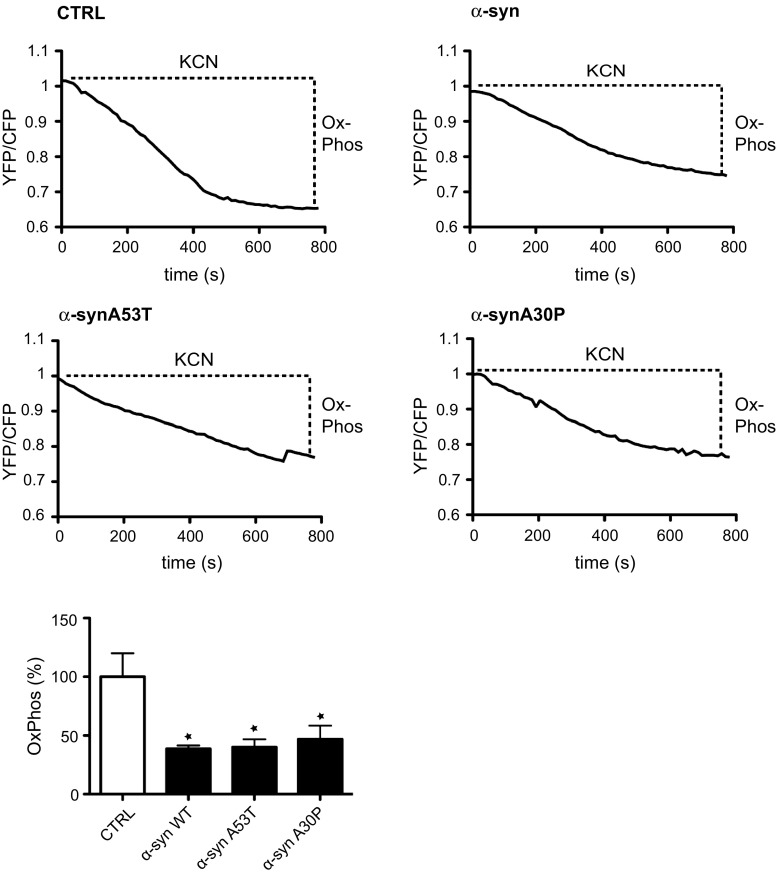
Reduced mitochondrial ATP production in rat cortical neurons expressing α-synuclein, α-synucleinA53T or α-synucleinA30P. 7-day-old rat neurons were co-transfected with the ATP indicator AT1.03 and either control vector, α-synuclein, α-synucleinA53T or α-synucleinA30P. Cells were imaged in time-lapse and treated with KCN to inhibit oxidative phosphorylation. Representative traces of YFP/CFP ratios are shown for the different samples. The fall in YFP/CFP ratios correlates with ATP produced by oxidative phosphorylation. *Bar chart* shows relative ATP levels produced by oxidative phosphorylation (OxPhos) in the different samples. Data were analysed by one-way ANOVA and Tukey’s post hoc test. *N* = 4–6, *error bars* are SEM; *p < 0.05

To complement these studies, we also used a FRET reporter system that permits ATP quantification in single living transfected cells [42]. Cellular ATP is generated by a combination of oxidative phosphorylation and glycolysis and so we assayed ATP production in cells treated with potassium cyanide (KCN) which inhibits cytochrome C oxidase to block oxidative phosphorylation. Monitoring ATP levels in the individually transfected cells prior to and after KCN treatment thus permits calculation of the levels of mitochondrial ATP production [42, 79]. SH-SY5Y cells were, therefore, co-transfected with the ATP FRET reporter and either control vector, wild-type or mutant α-synuclein and the relative ATP levels generated by oxidative phosphorylation determined. These studies revealed that compared to control transfected cells, both wild-type and mutant α-synuclein reduced mitochondrial ATP production (Fig. 6c). We also used the ATP FRET reporter to quantify mitochondrial ATP production in rat cortical neurons co-transfected with control vector, wild-type or mutant α-synuclein. These experiments revealed that α-synuclein likewise reduced mitochondrial ATP production in the neurons (Fig. 7). Thus, the α-synuclein-induced disruptions to IP3 receptor-mediated Ca^2+^ delivery to mitochondria are accompanied by reductions in mitochondrial ATP production.

### α-Synuclein does not cause a noticeable increase in GSK-3β activity

Recently, GSK-3β has been shown to regulate ER–mitochondria associations and binding of VAPB to PTPIP51 [78, 79]. Inhibition of GSK-3β promotes whereas activation inhibits the VAPB-PTPIP51 interaction and this leads to complementary changes in ER–mitochondria associations [78, 79]. The α-synuclein-induced loosening of ER–mitochondria associations and the VAPB–PTPIP51 interaction may, therefore, involve activation of GSK-3β. A major route for regulating GSK-3β activity involves inhibitory phosphorylation of serine-9 [44] and so we monitored GSK-3β serine-9 phosphorylation in control, α-synuclein, α-synucleinA53T and α-synucleinA30P expressing SH-SY5Y cells by immunoblotting. However, we detected no differences in GSK-3β serine-9 phosphorylation between these different cells (Supplemental Fig. 3).

### α-Synuclein is present in MAM and binds to VAPB

**Fig. 8:**
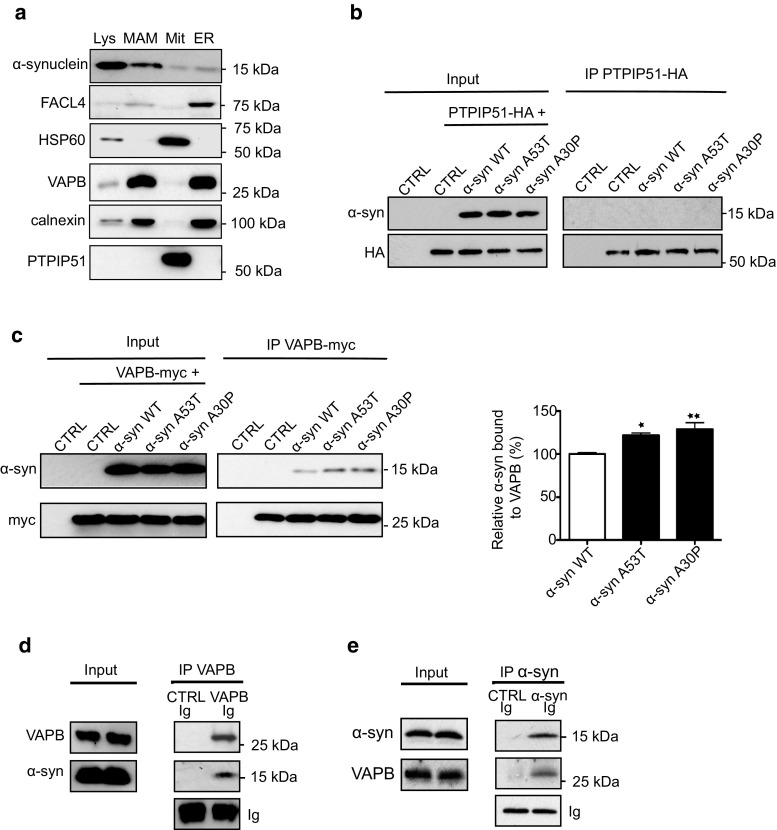
α-Synuclein is a MAM protein and binds to VAPB but not PTPIP51 in immunoprecipitation assays. **a** Immunoblots of total lysates (Lys), MAM, mitochondria (Mit) and ER proteins from rat brains. Samples were probed for α-synuclein plus FACL4, VAPB and calnexin as MAM/ER markers, and PTPIP51 and HSP60 as mitochondrial markers. Extended exposures of immunoblots reveal PTPIP51 signal in total lysate samples. **b**, **c** Immunoprecipitation assays of α-synuclein binding to PTPIP51 and VAPB. HEK293 cells were transfected with either PTPIP51-HA (**b**) or myc-VAPB (**c**) + either empty control vector (CTRL), α-synuclein, α-synucleinA53T or α-synucleinA30P as indicated. PTPIP51 was immunoprecipitated using rabbit anti-HA and detected on immunoblots with mouse anti-HA; α-synuclein was detected with mouse anti-α-synuclein. VAPB was immunoprecipitated using mouse anti-myc and detected on immunoblots with rabbit anti-HA; α-synuclein was detected with rabbit anti-α-synuclein. Both inputs and immunoprecipitations (IP) are shown. α-Synuclein displayed no binding to PTPIP51 but bound to VAPB. *Bar chart* in **c** shows relative levels of α-synuclein bound to VAPB in the immunoprecipitations following quantification of signals from immunoblots. α-Synuclein signals were normalized to immunoprecipitated VAPB-myc signals. Data are expressed as percentage of the wild-type α-synuclein signal and were analysed by one-way ANOVA and Tukey’s post hoc test. *N* = 4; *error bars* are SEM, *p > 0.05, **p < 0.01. **d**, **e** Endogenous VAPB and α-synuclein bind in immunoprecipitation assays from rat brain. **d** VAPB was immunoprecipitated using rabbit anti-VAPB and detected with rat anti-VAPB; α-synuclein was detected with mouse anti-α-synuclein. **e** α-Synuclein was immunoprecipitated using mouse anti-α-synuclein and detected using rabbit anti-α-synuclein; VAPB was detected using rabbit anti-VAPB. Control immunoprecipitations were performed with pre-immune serum or normal mouse Igs. Both inputs and immunoprecipitations (IP) are shown along with immunoglobulin heavy chain signals (Ig) to demonstrate equal Ig amounts in the VAPB and control immunoprecipitations. Molecular masses on immunoblots in kD are shown on the right

An alternative mechanism whereby α-synuclein could disrupt the VAPB-PTPIP51 tethers might involve its binding to either VAPB or PTPIP51 so as to sterically interfere with their interaction. In support of this notion, a proportion of α-synuclein localizes to MAM [31, 67]. To test this possibility further, we first sought to confirm the biochemical localization of α-synuclein to MAM and so prepared MAM, mitochondria and ER fractions from rat brain, and probed these for the presence of α-synuclein on immunoblots. In agreement with earlier studies [31], we detected a significant proportion of α-synuclein in MAM (Fig. 8a). We, therefore, monitored binding of wild-type and mutant α-synuclein to PTPIP51 and VAPB using immunoprecipitation assays from PTPIP51 + α-synuclein and VAPB + α-synuclein co-transfected cells. Although we detected no interaction between α-synuclein and PTPIP51 in these assays, we obtained robust signals for binding of both wild-type and mutant α-synuclein to VAPB (Fig. 8b, c). Moreover, we obtained stronger signals for binding of VAPB to α-synucleinA53T and α-synucleinA30P than to wild-type α-synuclein (Fig. 8c). We also used immunoprecipitation assays from rat brain to determine whether endogenous α-synuclein and VAPB interact. Again, α-synuclein bound to VAPB in these assays (Fig. 8d, e).

**Fig. 9:**
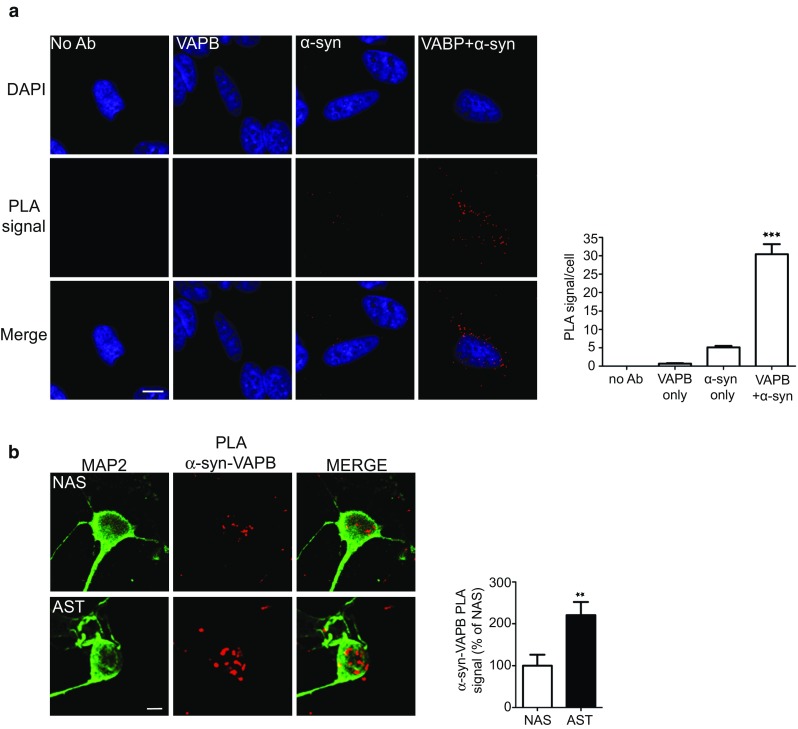
α-Synuclein binds to VAPB in proximity ligation assays (PLAs). Assays were performed with rat anti-VAPB and rabbit α-synuclein antibodies. **a** Endogenous α-synuclein and VAPB interact in SH-SY5Y cells. Control assays involving no primary antibodies (No Ab), VAPB or α-synuclein only antibodies produced very few signals but inclusion of both VAPB and α-synuclein antibodies generated robust signals. Representative images are shown and cells were stained with DAPI to show nuclei. *Scale bar* is 15 μm. *Bar chart* shows proximity ligation assay signals/cell in the different samples. Data were analysed by one-way ANOVA and Tukey’s post hoc test; *error bars* are SEM. *N* = 41–54 cells; ***p < 0.001. **b** Endogenous α-synuclein and VAPB interact in human iPS cell derived neurons and increased binding is seen in α-synuclein *SNCA* triplication (AST) cells compared to control (NAS) cells. Representative images are shown and cells were stained with MAP2 to show processes. *Scale bar* is 10 μm. *Bar chart* shows quantification of proximity ligation assay signals in the samples. Data were analysed by Student’s *t* test. *N* = 22–32 cells from 3 different experiments. **p < 0.01, *error bars* are SEM

To confirm the binding of VAPB to α-synuclein using other methods, we first performed proximity ligation assays in non-transfected SH-SY5Y cells using VAPB and α-synuclein antibodies. Omission of primary VAPB and/or α-synuclein antibodies produced very few signals but inclusion of both antibodies generated robust signals consistent with a direct interaction of the two endogenous proteins in situ (Fig. 9a). We also performed these assays in the control (NAS) and α-synuclein triplication (AST) iPS cell neurons and these likewise produced signals consistent with a direct interaction (Fig. 9b). Moreover, we obtained greater numbers of signals in the AST neurons that express higher levels of α-synuclein [21] (Fig. 9b).

**Fig. 10:**
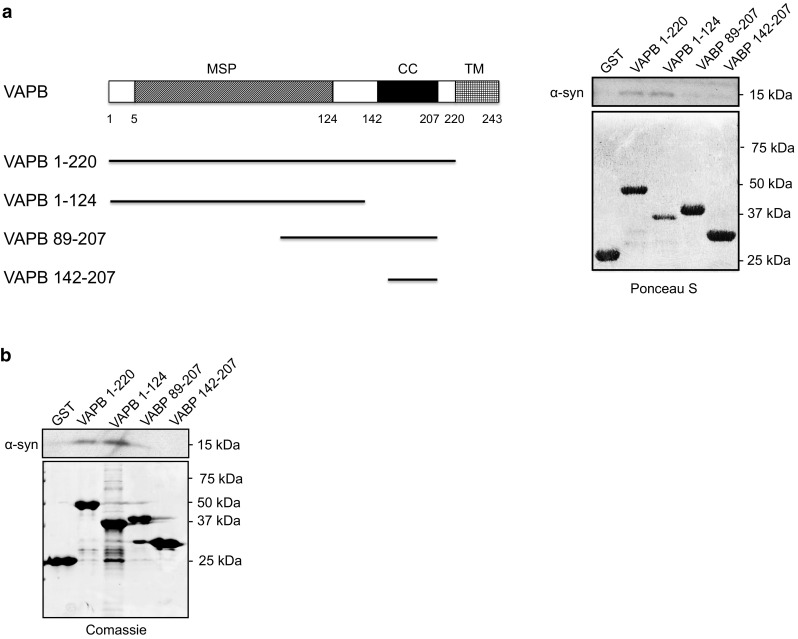
α-Synuclein binds to VAPB in GST pull-down assays and binding involves the VAPB MSP domain. **a** In vitro binding of recombinant α-synuclein to VAPB. Structure of VAPB (not to scale) with the MSP, coiled-coil (CC) and transmembrane (TM) domains illustrated along with GST-VAPB baits used in the pull-down assays are shown on the left. Ponceau S stained immunoblot of control GST and GST-VAPB baits (lower) and immunoblot showing bound α-synuclein (upper) are shown on the right. **b** Cellular binding of α-synuclein to VAPB in GST pull-down assays. GST and GST-VAPB baits were used in pull-down assays from HEK293 cells transfected with α-synuclein. Coomassie stained gel of GST baits (lower) and immunoblot showing bound α-synuclein (upper) are shown. Molecular masses in kD are shown on the right

To test this direct binding further, we prepared recombinant VAPB and α-synuclein in *E. coli* and monitored their binding in vitro. For these experiments, we generated different domains of VAPB as GST fusion proteins and used the GST moiety to isolate the VAPB “baits” along with any bound α-synuclein. VAPB is anchored in the ER via a C-terminal membrane-spanning domain with its N-terminus projecting into the cytoplasm through which it interacts with PTPIP51 [20, 78]. The VAPB cytoplasmic region contains an N-terminal major sperm protein (MSP) domain and a centrally located coiled-coil domain (Fig. 10a). We, therefore, prepared GST-VAPB “baits” comprising the entire cytoplasmic domain, the MSP domain, the coiled-coil domain and sequences encompassing the C-terminal cytosolic region of VAPB, and tested their abilities to bind to α-synuclein in pull-down assays. Both the entire VAPB cytoplasmic domain and the MSP domain, but not the coiled-coil or C-terminal domains bound to α-synuclein in these assays (Fig. 10a). Finally, we used the GST-VAPB “baits” to pull down α-synuclein from transfected cell lysates. Again, only the entire VAPB cytoplasmic domain and the MSP domain bound to VAPB in these cellular assays. Thus, VAPB and α-synuclein interact in a variety of assays including in vitro assays in the absence of other proteins and this interaction involves the VAPB MSP domain.

## Discussion

Despite the wealth of data linking α-synuclein to Parkinson’s disease, the targets for α-synuclein toxicity are not fully understood. Here, we show that α-synuclein perturbs ER–mitochondria associations and that this involves disruption to the VAPB-PTPIP51 tethering proteins. Importantly, this damage is also seen in iPS cell-derived dopaminergic neurons from Parkinson’s disease patients harbouring triplication of the α-synuclein locus. Although we used different α-synuclein triplication and control iPS cell clones in these latter studies, it will be important to confirm in future studies that other genetic differences between the clones do not contribute to this phenotype. This could be achieved by analyses of genetically corrected mutant clones such that the genetic backgrounds are identical. Using a range of assays including immunoprecipitation, cellular GST pull-down, proximity ligation and in vitro binding of recombinant proteins, we also show that α-synuclein is a direct binding partner for VAPB. Interestingly, earlier mass spectrometry proteomic analyses also suggested that α-synuclein was complexed with VAPB although these studies did not discriminate between direct and indirect binding nor provide confirmatory data [58].

A primary function of ER–mitochondria associations is to deliver Ca^2+^ from ER stores to mitochondria. This delivery involves release of Ca^2+^ from IP3 receptors in MAM and uptake via the mitochondrial voltage dependent anion channel (VDAC) [46, 48, 63, 70, 82]. Mitochondria require Ca^2+^ to efficiently produce ATP since several dehydrogenases in the tricarboxylic acid cycle are Ca^2+^ regulated [30]. Thus, disruption to the VAPB-PTPIP51 tethers perturbs IP3 receptor-mediated ER–mitochondria Ca^2+^ exchange and mitochondrial ATP production [20, 78, 79]. Consistent with these findings, we show that disruption of the VAPB-PTPIP51 tethers by α-synuclein is accompanied by reductions in IP3 receptor-mediated Ca^2+^ delivery to mitochondria. We also show that there are reductions in mitochondrial ATP production in α-synuclein expressing cells which is in line with reduced mitochondrial Ca^2+^ levels. However, α-synuclein has been shown to damage a number of mitochondrial proteins that could impact upon ATP production. These include VDAC, mitochondrial ATP synthase and TOM20 [22, 56, 69]. Thus, there may be several routes including the VAPB-PTPIP51 tethers by which α-synuclein could deleteriously affect mitochondrial function and ATP production, and the precise contributions of each of these are not as yet clear.

Neurons are particularly dependent upon correct Ca^2+^ signalling since it is involved in depolarization and synaptic activity [10]. Neurons also consume large amounts of energy [50]. Thus changes to Ca^2+^ signalling and mitochondrial ATP production are strongly implicated in Parkinson’s disease and other neurodegenerative diseases [2, 10, 14, 62, 65]. Indeed, elegant molecular modelling studies have shown that even relatively small reductions in mitochondrial ATP production can be sufficient to induce many salient features of neurodegenerative diseases [51]. The α-synuclein induced disruptions to ER–mitochondria Ca^2+^ exchange and mitochondrial ATP production that we describe here are thus likely to be major drivers of disease.

Changes in mitochondrial morphology have been associated with α-synuclein [8] and there is evidence of mitochondrial “rounding up” and clustering in our α-synuclein expressing cells. Whether such morphological alterations are linked to the changes in ER–mitochondria contacts that we describe are not clear. We did not detect any gross changes to ER morphology. However, several recent studies have shown that ER–mitochondria contact sites regulate mitochondrial biogenesis, division and DNA synthesis [25, 47, 53]. One possibility is that the effects of α-synuclein on mitochondrial morphology are linked to its function at ER–mitochondria contact sites.

In immunoprecipitation assays, we found that whilst both wild-type and mutant α-synuclein bound to VAPB, the mutants bound slightly stronger. However, in the functional assays involving ER–mitochondria Ca^2+^ exchange and mitochondrial ATP production we did not detect robust differences between wild-type and mutant α-synuclein. This may be due to the sensitivity of these assays in detecting changes induced by relatively small alterations in binding of wild-type and mutant α-synuclein to VAPB. However, our findings are in line with human disease phenotypes. Triplication of the α-synuclein gene leading to increased expression of wild-type α-synuclein is pathogenic but the familial mutants are not associated with such overexpression. The increased binding of mutant α-synuclein to VAPB, therefore, provides a possible explanation for the similar pathogenic effects of wild-type and mutant α-synuclein involving the VAPB-PTPIP51 tethers. Thus, triplication generates increased α-synuclein which binds to VAPB to disrupt the VAPB-PTPIP51 tethers. Mutant α-synuclein expressed at normal levels binds slightly stronger to VAPB to equally disrupt the VAPB-PTPIP51 tethers.

Two other studies have investigated the effect of α-synuclein on ER–mitochondria associations but the findings are inconsistent [11, 31]. In particular, one reports that wild-type and mutant α-synuclein reduce whereas the other reports that wild-type increases contacts [11, 31]. The reasons for these different findings are not clear but both studies included confocal microscopy experiments to quantify ER–mitochondria associations. ER–mitochondria contacts are defined as involving 10–30 nm distances which is at least an order of magnitude beyond the resolution of the confocal microscope; appropriate microscopy methods, therefore, need to be used when quantifying contacts [16, 37, 55, 63]. Here, we utilized EM, proximity ligation assays and super resolution SIM methods to quantify ER–mitochondria contacts and all revealed that expression of wild-type and mutant α-synuclein decrease ER–mitochondria contacts. Such methods afford better resolution for properly quantifying ER–mitochondria associations. We also found that α-synuclein disrupts binding between the ER–mitochondria tethering proteins VAPB and PTPIP51. Finally, we show that α-synuclein expression perturbs Ca^2+^ uptake by mitochondria following IP3 receptor-mediated release from ER stores which is a physiological readout of ER–mitochondria associations. Together, these findings using different but complementary methods and approaches demonstrate that overexpression of α-synuclein disrupts ER–mitochondria contacts.

Recently, Tar DNA-binding protein-43 (TDP-43) and Fused in Sarcoma (FUS), two proteins intimately linked to fronto-temporal dementia and related amyotrophic lateral sclerosis (FTD/ALS) have also been shown to disrupt ER–mitochondria associations [78, 79]. As is the case with α-synuclein, these effects of TDP-43 and FUS involve breaking of the VAPB-PTPIP51 tethers. However, for TDP-43 and FUS, this breaking involves activation of GSK-3β; GSK-3β is a regulator of the VAPB–PTPIP51 interaction and so controls ER–mitochondria associations and Ca^2+^ exchange [78, 79]. GSK-3β has been linked to α-synuclein and Parkinson’s disease [27, 54, 57] but we found no evidence that either wild-type or mutant α-synuclein expression caused activation of GSK-3β. Rather, we found that α-synuclein bound directly to VAPB. Thus, there appears to be different routes by which neurodegenerative disease insults can impact upon ER–mitochondria tethering via the VAPB–PTPIP51 interaction, some involving activation of GSK-3β and some such as we describe here for α-synuclein, involving binding to the tethering proteins.

α-Synuclein toxicity has been linked to a number of seemingly diverse pathological features in Parkinson’s disease. These include damage to mitochondria, the ER, axonal transport, autophagy, Ca^2+^ homeostasis and lipid metabolism [9, 11, 13, 22, 31, 32, 38, 59, 60, 68, 71, 84, 88, 90, 91]. Indeed, the difficulty in deciphering α-synuclein toxicity is linking these different pathological changes to a common disease pathway. However, all of these physiological processes are regulated by signalling between ER and mitochondria at MAM [46, 48, 63, 70, 82, 83]. Thus, our demonstration that α-synuclein binds to VAPB, disrupts the VAPB-PTPIP51 tethers, ER–mitochondria contacts and signalling, represents a plausible route whereby α-synuclein may damage such a variety of cellular functions. These molecular findings facilitate a proper dissection of the role of ER–mitochondria signaling in α-synuclein linked Parkinson’s disease.

## Electronic supplementary material

Below is the link to the electronic supplementary material.

Supplemental Fig. 1 Mitochondria and ER morphology changes in α-synuclein stably transfected SH-SY5Y cells. **a** Expression of EGFP-α-synuclein, EGFP-α-synucleinA53T or EGFP-α-synucleinA30P do not induce changes in the levels of PDI (ER) or TOM20 (mitochondria) compared to EGFP control (CRTL) cells. Cell samples were probed on immunoblots; GAPDH is shown as a loading control. Molecular masses in kD are shown on the right. **b** Increased mitochondrial circularity in EGFP-α-synuclein and EGFP-α-synucleinA53T cells, EGFP-α-synucleinA30P cells also showed a trend for increased circularity but this did not reach significance; *N* = 30. **c** Decreased mitochondrial cytosolic distribution (increased mitochondrial clustering) in EGFP-α-synuclein, EGFP-α-synucleinA53T and EGFP-α-synucleinA30P cells; *N* = 30. **d**, **e**, **f** No changes in ER cytosolic distributions, numbers of branch points or branch lengths in EGFP-α-synuclein, EGFP-α-synucleinA53T or EGFP-α-synucleinA30P cells; *N* = 10. Data were analysed by one-way ANOVA and Tukey’s post hoc test. *Error bars* are SEM; *p < 0.05, **p < 0.01, ***p < 0.001. NS, not significant (PDF 247 kb)

Supplemental Fig. 2 Co-localisation of α-synuclein with MAM at ER–mitochondria contacts in SH-SY5Y cells expressing wild-type or mutant α-synuclein. SH-SY5Y cells were transfected with either EGFP control vector (CTRL), EGFP-α-synuclein (α-syn), EGFP-α-synucleinA53T (A53T) or EGFP-α-synucleinA30P (A30P) and immunostained for PDI and TOM20 to label ER and mitochondria (Mito), respectively; α-synuclein were detected via their EGFP tags. PDI and TOM20 co-localisation signals (i.e. ER–mitochondria contacts at MAM) were then compared with α-synuclein signals and α-synuclein/MAM co-localisation signals (*far right panels*) displayed. A proportion of α-synuclein locates to ER–mitochondria contacts. *Scale bar* is 15 μm (PDF 607 kb)

Supplemental Fig. 3 Overexpression of α-synuclein does not activate GSK3β. Immunoblots of SH-SY5Y cells expressing control empty vector, wild-type α-synuclein, α-synucleinA53T or α-synucleinA30P probed for inactive GSK3β phosphorylated on serine-9 (GSK3β-P), total GSK3β, α-synuclein and GAPDH as a loading control. Molecular masses in kD are shown on the right. *Bar chart* shows GSK3β serine-9 phosphorylation signals normalized to controls. Data were analysed by one-way ANOVA. *N* = 4; *error bars* are SEM, *N.S.* not significant (PDF 152 kb)

## References

[1] Appel-Cresswell S, Vilarino-Guell C, Encarnacion M, Sherman H, Yu I, Shah B, Weir D, Thompson C, Szu-Tu C, Trinh J (2013). Alpha-synuclein p. H50Q, a novel pathogenic mutation for Parkinson’s disease. Mov Disord.

[2] Arduino DM, Esteves AR, Oliveira CR, Cardoso SM (2010). Mitochondrial metabolism modulation: a new therapeutic approach for Parkinson’s disease. CNS Neurol Disord: Drug Targets.

[3] Arganda-Carreras I, Fernandez-Gonzalez R, Munoz-Barrutia A, Ortiz-De-Solorzano C (2010). 3D reconstruction of histological sections: application to mammary gland tissue. Microsc Res Tech.

[4] Bartolome F, Wu HC, Burchell VS, Preza E, Wray S, Mahoney CJ, Fox NC, Calvo A, Canosa A, Moglia C (2013). Pathogenic VCP mutations induce mitochondrial incoupling and reduced ATP levels. Neuron.

[5] Bernard-Marissal N, Medard JJ, Azzedine H, Chrast R (2015). Dysfunction in endoplasmic reticulum–mitochondria crosstalk underlies SIGMAR1 loss of function mediated motor neuron degeneration. Brain.

[6] Bezard E, Yue Z, Kirik D, Spillantini MG (2013). Animal models of Parkinson’s disease: limits and relevance to neuroprotection studies. Mov Disord.

[7] Blandini F, Armentero MT (2012). Animal models of Parkinson’s disease. FEBS J.

[8] Bose A, Beal MF (2016). Mitochondrial dysfunction in Parkinson’s disease. J Neurochem.

[9] Breydo L, Wu JW, Uversky VN (2012). Alpha-synuclein misfolding and Parkinson’s disease. Biochim Biophys Acta.

[10] Brini M, Cali T, Ottolini D, Carafoli E (2014). Neuronal calcium signaling: function and dysfunction. Cell Mol Life Sci.

[11] Cali T, Ottolini D, Negro A, Brini M (2012). Alpha-synuclein controls mitochondrial calcium homeostasis by enhancing endoplasmic reticulum–mitochondria interactions. J Biol Chem.

[12] Chartier-Harlin MC, Kachergus J, Roumier C, Mouroux V, Douay X, Lincoln S, Levecque C, Larvor L, Andrieux J, Hulihan M (2004). Alpha-synuclein locus duplication as a cause of familial Parkinson’s disease. Lancet.

[13] Chung CY, Koprich JB, Siddiqi H, Isacson O (2009). Dynamic changes in presynaptic and axonal transport proteins combined with striatal neuroinflammation precede dopaminergic neuronal loss in a rat model of AAV alpha-synucleinopathy. J Neurosci.

[14] Correia SC, Santos RX, Perry G, Zhu X, Moreira PI, Smith MA (2012). Mitochondrial importance in Alzheimer’s, Huntington’s and Parkinson’s diseases. Adv Exp Med Biol.

[15] Cosson P, Marchetti A, Ravazzola M, Orci L (2012). Mitofusin-2 independent juxtaposition of endoplasmic reticulum and mitochondria: an ultrastructural study. PLoS ONE.

[16] Csordas G, Renken C, Varnai P, Walter L, Weaver D, Buttle KF, Balla T, Mannella CA, Hajnoczky G (2006). Structural and functional features and significance of the physical linkage between ER and mitochondria. J Cell Biol.

[17] Dagda RK, Cherra SJ, Kulich SM, Tandon A, Park D, Chu CT (2009). Loss of PINK1 function promotes mitophagy through effects on oxidative stress and mitochondrial fission. J Biol Chem.

[18] Dawson TM, Ko HS, Dawson VL (2010). Genetic animal models of Parkinson’s disease. Neuron.

[19] de Brito OM, Scorrano L (2008). Mitofusin 2 tethers endoplasmic reticulum to mitochondria. Nature.

[20] De Vos KJ, Morotz GM, Stoica R, Tudor EL, Lau KF, Ackerley S, Warley A, Shaw CE, Miller CCJ (2012). VAPB interacts with the mitochondrial protein PTPIP51 to regulate calcium homeostasis. Hum Mol Genet.

[21] Devine MJ, Ryten M, Vodicka P, Thomson AJ, Burdon T, Houlden H, Cavaleri F, Nagano M, Drummond NJ, Taanman JW (2011). Parkinson’s disease induced pluripotent stem cells with triplication of the alpha-synuclein locus. Nat Commun.

[22] Di Maio R, Barrett PJ, Hoffman EK, Barrett CW, Zharikov A, Borah A, Hu X, McCoy J, Chu CT, Burton EA (2016). alpha-Synuclein binds to TOM20 and inhibits mitochondrial protein import in Parkinson’s disease. Sci Transl Med.

[23] Filadi R, Greotti E, Turacchio G, Luini A, Pozzan T, Pizzo P (2015). Mitofusin 2 ablation increases endoplasmic reticulum–mitochondria coupling. Proc Natl Acad Sci USA.

[24] Filadi R, Greotti E, Turacchio G, Luini A, Pozzan T, Pizzo P (2016). Presenilin 2 modulates endoplasmic reticulum–mitochondria coupling by tuning the antagonistic effect of Mitofusin 2. Cell Rep.

[25] Friedman JR, Lackner LL, West M, Dibenedetto JR, Nunnari J, Voeltz GK (2011). ER tubules mark sites of mitochondrial division. Science.

[26] Galmes R, Houcine A, van Vliet AR, Agostinis P, Jackson CL, Giordano F (2016). ORP5/ORP8 localize to endoplasmic reticulum–mitochondria contacts and are involved in mitochondrial function. EMBO Rep.

[27] Golpich M, Amini E, Hemmati F, Ibrahim NM, Rahmani B, Mohamed Z, Raymond AA, Dargahi L, Ghasemi R, Ahmadiani A (2015). Glycogen synthase kinase-3 beta (GSK-3beta) signaling: implications for Parkinson’s disease. Pharmacol Res.

[28] Gomez-Suaga P, Paillusson S, Stoica R, Noble W, Hanger DP, Miller CC (2017). The ER–mitochondria tethering complex VAPB-PTPIP51 regulates autophagy. Curr Biol.

[29] Gregianin E, Pallafacchina G, Zanin S, Crippa V, Rusmini P, Poletti A, Fang M, Li Z, Diano L, Petrucci A (2016). Loss-of-function mutations in the SIGMAR1 gene cause distal hereditary motor neuropathy by impairing ER–mitochondria tethering and Ca2 + signalling. Hum Mol Genet.

[30] Griffiths EJ, Rutter GA (2009). Mitochondrial calcium as a key regulator of mitochondrial ATP production in mammalian cells. Biochim Biophys Acta.

[31] Guardia-Laguarta C, Area-Gomez E, Rub C, Liu Y, Magrane J, Becker D, Voos W, Schon EA, Przedborski S (2014). alpha-Synuclein is localized to mitochondria-associated ER membranes. J Neurosci.

[32] Guardia-Laguarta C, Area-Gomez E, Schon EA, Przedborski S (2015). Novel subcellular localization for alpha-synuclein: possible functional consequences. Front Neuroanat.

[33] Guerrero E, Vasudevaraju P, Hegde ML, Britton GB, Rao KS (2013). Recent advances in alpha-synuclein functions, advanced glycation, and toxicity: implications for Parkinson’s disease. Mol Neurobiol.

[34] Guidato S, McLoughlin DM, Grierson AJ, Miller CC (1998). Cyclin D2 interacts with cdk-5 and modulates cellular cdk-5/p35 activity. J Neurochem.

[35] Hansen C, Angot E, Bergstrom AL, Steiner JA, Pieri L, Paul G, Outeiro TF, Melki R, Kallunki P, Fog K (2011). alpha-Synuclein propagates from mouse brain to grafted dopaminergic neurons and seeds aggregation in cultured human cells. J Clin Invest.

[36] Hedskog L, Pinho CM, Filadi R, Ronnback A, Hertwig L, Wiehager B, Larssen P, Gellhaar S, Sandebring A, Westerlund M (2013). Modulation of the endoplasmic reticulum–mitochondria interface in Alzheimer’s disease and related models. Proc Natl Acad Sci USA.

[37] Helle SC, Kanfer G, Kolar K, Lang A, Michel AH, Kornmann B (2013). Organization and function of membrane contact sites. Biochim Biophys Acta.

[38] Hettiarachchi NT, Parker A, Dallas ML, Pennington K, Hung CC, Pearson HA, Boyle JP, Robinson P, Peers C (2009). alpha-Synuclein modulation of Ca^2+^ signaling in human neuroblastoma (SH-SY5Y) cells. J Neurochem.

[39] Hirano Y, Matsuda A, Hiraoka Y (2015). Recent advancements in structured-illumination microscopy toward live-cell imaging. Microscopy (Oxf).

[40] Huttlin EL, Ting L, Bruckner RJ, Gebreab F, Gygi MP, Szpyt J, Tam S, Zarraga G, Colby G, Baltier K (2015). The BioPlex network: a systematic exploration of the human interactome. Cell.

[41] Ibanez P, Bonnet AM, Debarges B, Lohmann E, Tison F, Pollak P, Agid Y, Durr A, Brice A (2004). Causal relation between alpha-synuclein gene duplication and familial Parkinson’s disease. Lancet.

[42] Imamura H, Nhat KP, Togawa H, Saito K, Iino R, Kato-Yamada Y, Nagai T, Noji H (2009). Visualization of ATP levels inside single living cells with fluorescence resonance energy transfer-based genetically encoded indicators. Proc Natl Acad Sci USA.

[43] Jakes R, Spillantini MG, Goedert M (1994). Identification of two distinct synucleins from human brain. FEBS Lett.

[44] Kaidanovich-Beilin O, Woodgett JR (2011). GSK-3: functional insights from cell biology and animal models. Front Mol Neurosci.

[45] Kiely AP, Asi YT, Kara E, Limousin P, Ling H, Lewis P, Proukakis C, Quinn N, Lees AJ, Hardy J (2013). alpha-Synucleinopathy associated with G51D SNCA mutation: a link between Parkinson’s disease and multiple system atrophy?. Acta Neuropathol.

[46] Kornmann B (2013). The molecular hug between the ER and the mitochondria. Curr Opin Cell Biol.

[47] Korobova F, Ramabhadran V, Higgs HN (2013). An actin-dependent step in mitochondrial fission mediated by the ER-associated formin INF2. Science.

[48] Krols M, van Isterdael G, Asselbergh B, Kremer A, Lippens S, Timmerman V, Janssens S (2016). Mitochondria-associated membranes as hubs for neurodegeneration. Acta Neuropathol.

[49] Krüger R, Kuhn W, Müller T, Woitalla D, Graeber M, Kösel S, Przuntek H, Epplen JT, Schöls L, Riess O (1998). Ala30Pro mutation in the gene encoding α-synuclein in Parkinson’s disease. Nature Genet.

[50] Laughlin SB, de Ruyter van Steveninck RR, Anderson JC (1998). The metabolic cost of neural information. Nat Neurosci.

[51] Le Masson G, Przedborski S, Abbott LF (2014). A computational model of motor neuron degeneration. Neuron.

[52] Leal NS, Schreiner B, Pinho CM, Filadi R, Wiehager B, Karlstrom H, Pizzo P, Ankarcrona M (2016). Mitofusin-2 knockdown increases ER–mitochondria contact and decreases amyloid beta-peptide production. J Cell Mol Med.

[53] Lewis SC, Uchiyama LF, Nunnari J (2016). ER–mitochondria contacts couple mtDNA synthesis with mitochondrial division in human cells. Science.

[54] Li DW, Liu ZQ, Chen W, Yao M, Li GR (2014). Association of glycogen synthase kinase-3beta with Parkinson’s disease (review). Mol Med Rep.

[55] Lidke DS, Lidke KA (2012). Advances in high-resolution imaging—techniques for three-dimensional imaging of cellular structures. J Cell Sci.

[56] Ludtmann MH, Angelova PR, Ninkina NN, Gandhi S, Buchman VL, Abramov AY (2016). Monomeric alpha-synuclein exerts a physiological role on brain ATP synthase. J Neurosci.

[57] Majd S, Power JH, Grantham HJ (2015). Neuronal response in Alzheimer’s and Parkinson’s disease: the effect of toxic proteins on intracellular pathways. BMC Neurosci.

[58] McFarland MA, Ellis CE, Markey SP, Nussbaum RL (2008). Proteomics analysis identifies phosphorylation-dependent alpha-synuclein protein interactions. Mol Cell Proteomics.

[59] Mercado G, Valdes P, Hetz C (2013). An ERcentric view of Parkinson’s disease. Trends Mol Med.

[60] Nakamura K, Nemani VM, Azarbal F, Skibinski G, Levy JM, Egami K, Munishkina L, Zhang J, Gardner B, Wakabayashi J (2011). Direct membrane association drives mitochondrial fission by the Parkinson disease-associated protein alpha-synuclein. J Biol Chem.

[61] Nath S, Goodwin J, Engelborghs Y, Pountney DL (2011). Raised calcium promotes alpha-synuclein aggregate formation. Mol Cell Neurosci.

[62] Nicholls DG (2008). Oxidative stress and energy crises in neuronal dysfunction. Ann N Y Acad Sci.

[63] Paillusson S, Stoica R, Gomez-Suaga P, Lau DH, Mueller S, Miller T, Miller CC (2016). There’s something wrong with my MAM—the ER–mitochondria axis and neurodegenerative diseases. Trends Neurosci.

[64] Pandey N, Schmidt RE, Galvin JE (2006). The alpha-synuclein mutation E46K promotes aggregation in cultured cells. Exp Neurol.

[65] Pilsl A, Winklhofer KF (2012). Parkin, PINK1 and mitochondrial integrity: emerging concepts of mitochondrial dysfunction in Parkinson’s disease. Acta Neuropathol.

[66] Polymeropoulos MH, Lavedan C, Leroy E, Ide SE, Dehejia A, Dutra A, Pike B, Root H, Rubenstein J, Boyer R (1997). Mutation in the α-synuclein gene identified in families with Parkinson’s disease. Science.

[67] Poston CN, Krishnan SC, Bazemore-Walker CR (2013). In-depth proteomic analysis of mammalian mitochondria-associated membranes (MAM). J Proteomics.

[68] Prots I, Veber V, Brey S, Campioni S, Buder K, Riek R, Bohm KJ, Winner B (2013). Alpha-synuclein oligomers impair neuronal microtubule-kinesin interplay. J Biol Chem.

[69] Rostovtseva TK, Gurnev PA, Protchenko O, Hoogerheide DP, Yap TL, Philpott CC, Lee JC, Bezrukov SM (2015). alpha-Synuclein shows high affinity interaction with Voltage-dependent Anion Channel, suggesting mechanisms of mitochondrial regulation and toxicity in Parkinson Disease. J Biol Chem.

[70] Rowland AA, Voeltz GK (2012). Endoplasmic reticulum–mitochondria contacts: function of the junction. Nat Rev Mol Cell Biol.

[71] Saha AR, Hill J, Utton MA, Asuni AA, Ackerley S, Grierson AJ, Miller CC, Davies AM, Buchman VL, Anderton BH (2004). Parkinson’s disease alpha-synuclein mutations exhibit defective axonal transport in cultured neurons. J Cell Sci.

[72] Schwach G, Tschemmernegg M, Pfragner R, Ingolic E, Schreiner E, Windisch M (2010). Establishment of stably transfected rat neuronal cell lines expressing alpha-synuclein GFP fusion proteins. J Mol Neurosci.

[73] Shaltiel-Karyo R, Frenkel-Pinter M, Egoz-Matia N, Frydman-Marom A, Shalev DE, Segal D, Gazit E (2010). Inhibiting alpha-synuclein oligomerization by stable cell-penetrating beta-synuclein fragments recovers phenotype of Parkinson’s disease model flies. PLoS ONE.

[74] Singleton AB, Farrer M, Johnson J, Singleton A, Hague S, Kachergus J, Hulihan M, Peuralinna T, Dutra A, Nussbaum R (2003). alpha-Synuclein locus triplication causes Parkinson’s disease. Science.

[75] Soderberg O, Gullberg M, Jarvius M, Ridderstrale K, Leuchowius KJ, Jarvius J, Wester K, Hydbring P, Bahram F, Larsson LG (2006). Direct observation of individual endogenous protein complexes in situ by proximity ligation. Nat Methods.

[76] Spillantini MG, Schmidt ML, Lee VM-Y, Trojanowski JQ, Jakes R, Goedert M (1997). Alpha-synuclein in Lewy bodies. Nature.

[77] Spinelli KJ, Osterberg VR, Meshul CK, Soumyanath A, Unni VK (2015). Curcumin treatment improves motor behavior in alpha-synuclein transgenic mice. PLoS ONE.

[78] Stoica R, De Vos KJ, Paillusson S, Mueller S, Sancho RM, Lau KF, Vizcay-Barrena G, Lin WL, Xu YF, Lewis J (2014). ER–mitochondria associations are regulated by the VAPB-PTPIP51 interaction and are disrupted by ALS/FTD-associated TDP-43. Nat Commun.

[79] Stoica R, Paillusson S, Gomez-Suaga P, Mitchell JC, Lau DH, Gray EH, Sancho RM, Vizcay-Barrena G, De Vos KJ, Shaw CE (2016). ALS/FTD-associated FUS activates GSK-3beta to disrupt the VAPB-PTPIP51 interaction and ER–mitochondria associations. EMBO Rep.

[80] Takeda A, Mallory M, Sundsmo M, Honer W, Hansen L, Masliah E (1998). Abnormal accumulation of NACP/a-synuclein in neurodegenerative disorders. Am J Pathol.

[81] Vagnoni A, Perkinton MS, Gray EH, Francis PT, Noble W, Miller CCJ (2012). Calsyntenin-1 mediates axonal transport of the amyloid precursor protein and regulates Abeta production. Hum Mol Genet.

[82] van Vliet A, Verfaillie T, Agostinis P (2014). New functions of mitochondria associated membranes in cellular signalling. Biochim Biophys Acta.

[83] Vance JE (2015). Phospholipid synthesis and transport in mammalian cells. Traffic (Copenhagen, Denmark).

[84] Volpicelli-Daley LA, Gamble KL, Schultheiss CE, Riddle DM, West AB, Lee VM (2014). Formation of alpha-synuclein Lewy neurite-like aggregates in axons impedes the transport of distinct endosomes. Mol Biol Cell.

[85] Wang PT, Garcin PO, Fu M, Masoudi M, St-Pierre P, Pante N, Nabi IR (2015). Distinct mechanisms controlling rough and smooth endoplasmic reticulum–mitochondria contacts. J Cell Sci.

[86] Watanabe S, Ilieva H, Tamada H, Nomura H, Komine O, Endo F, Jin S, Mancias P, Kiyama H, Yamanaka K (2016). Mitochondria-associated membrane collapse is a common pathomechanism in SIGMAR1- and SOD1-linked ALS. EMBO Mol Med.

[87] Wieckowski MR, Giorgi C, Lebiedzinska M, Duszynski J, Pinton P (2009). Isolation of mitochondria-associated membranes and mitochondria from animal tissues and cells. Nat Protoc.

[88] Winslow AR, Chen CW, Corrochano S, Acevedo-Arozena A, Gordon DE, Peden AA, Lichtenberg M, Menzies FM, Ravikumar B, Imarisio S (2010). alpha-Synuclein impairs macroautophagy: implications for Parkinson’s disease. J Cell Biol.

[89] Wray S, Self M, Lewis PA, Taanman JW, Ryan NS, Mahoney CJ, Liang Y, Devine MJ, Sheerin UM, Houlden H (2012). Creation of an open-access, mutation-defined fibroblast resource for neurological disease research. PLoS ONE.

[90] Xie W, Chung KK (2012). Alpha-synuclein impairs normal dynamics of mitochondria in cell and animal models of Parkinson’s disease. J Neurochem.

[91] Xu W, Tan L, Yu JT (2015). The link between the SNCA gene and parkinsonism. Neurobiol Aging.

[92] Zarranz JJ, Alegre J, Gomez-Esteban JC, Lezcano E, Ros R, Ampuero I, Vidal L, Hoenicka J, Rodriguez O, Atares B (2004). The new mutation, E46K, of alpha-synuclein causes Parkinson and Lewy body dementia. Ann Neurol.

