# Supplementary figures and images for: α-Synuclein binds to the ER–mitochondria tethering protein VAPB to disrupt Ca2+ homeostasis and mitochondrial ATP production
Source: Acta Neuropathol. 2017 Mar 23;134(1):129–49. doi: 10.1007/s00401-017-1704-z (PMC5486644; doi:10.1007/s00401-017-1704-z)

Supplemental Figure 1

**a**

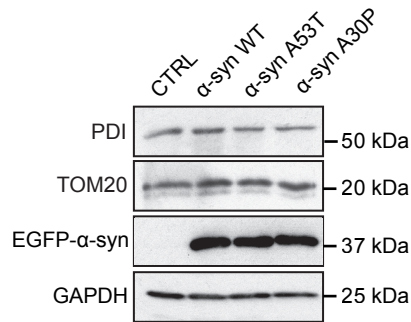

**b**

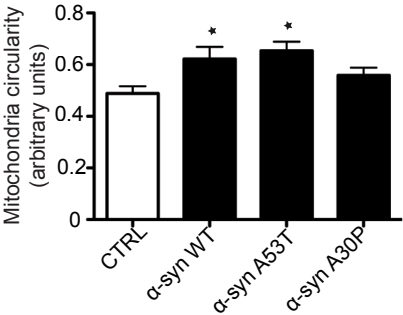

**c**

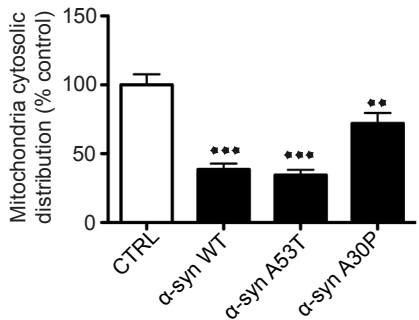

**d**

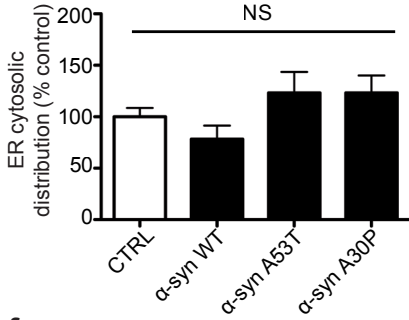

**e**

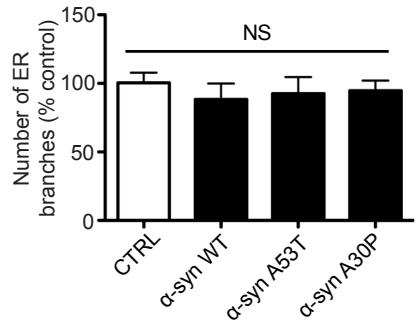

**f**

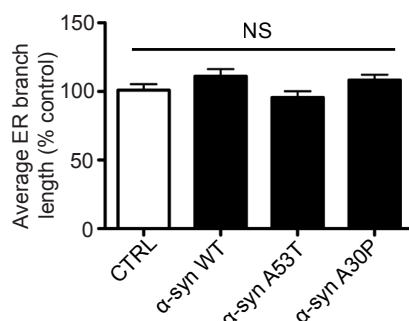

Supplement: Supplementary file 1 — Supplemental Fig. 1 Mitochondria and ER morphology changes in α-synuclein stably transfected SH-SY5Y cells. a Expression of EGFP-α-synuclein, EGFP-α-synucleinA53T or EGFP-α-synucleinA30P do not induce changes in the levels of PDI (ER) or TOM20 (mitochondria) compared to EGFP control (CRTL) cells. Cell samples were probed on immunoblots; GAPDH is shown as a loading control. Molecular masses in kD are shown on the right. b Increased mitochondrial circularity in EGFP-α-synuclein and EGFP-α-synucleinA53T cells, EGFP-α-synucleinA30P cells also showed a trend for increased circularity but this did not reach significance; N = 30. c Decreased mitochondrial cytosolic distribution (increased mitochondrial clustering) in EGFP-α-synuclein, EGFP-α-synucleinA53T and EGFP-α-synucleinA30P cells; N = 30. d, e, f No changes in ER cytosolic distributions, numbers of branch points or branch lengths in EGFP-α-synuclein, EGFP-α-synucleinA53T or EGFP-α-synucleinA30P cells; N = 10. Data were analysed by one-way ANOVA and Tukey’s post hoc test. Error bars are SEM; *p < 0.05, **p < 0.01, ***p < 0.001. NS, not significant (PDF 247 kb) [file 401_2017_1704_MOESM1_ESM.pdf]

Supplemental Figure 2

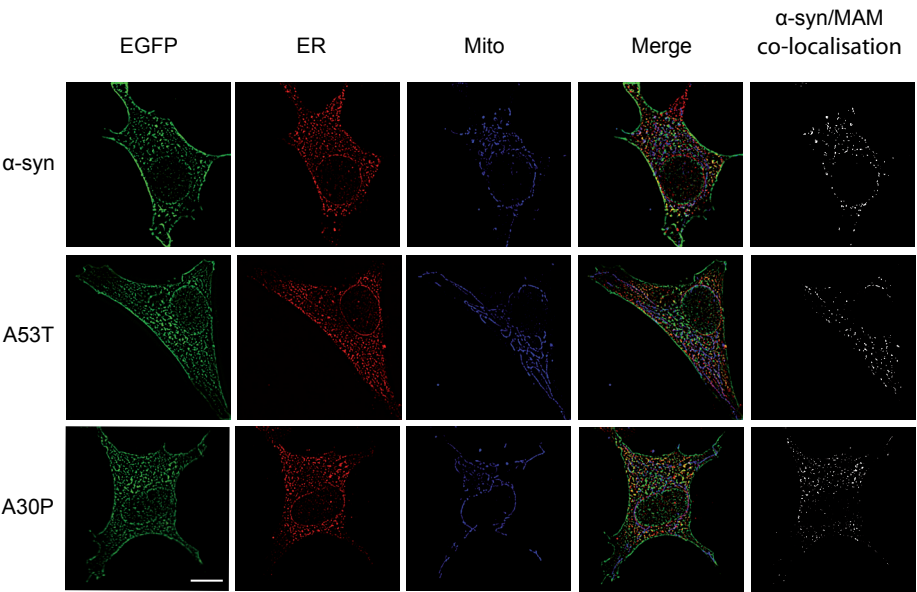

Supplement: Supplementary file 2 — Supplemental Fig. 2 Co-localisation of α-synuclein with MAM at ER–mitochondria contacts in SH-SY5Y cells expressing wild-type or mutant α-synuclein. SH-SY5Y cells were transfected with either EGFP control vector (CTRL), EGFP-α-synuclein (α-syn), EGFP-α-synucleinA53T (A53T) or EGFP-α-synucleinA30P (A30P) and immunostained for PDI and TOM20 to label ER and mitochondria (Mito), respectively; α-synuclein were detected via their EGFP tags. PDI and TOM20 co-localisation signals (i.e. ER–mitochondria contacts at MAM) were then compared with α-synuclein signals and α-synuclein/MAM co-localisation signals (far right panels) displayed. A proportion of α-synuclein locates to ER–mitochondria contacts. Scale bar is 15 μm (PDF 607 kb) [file 401_2017_1704_MOESM2_ESM.pdf]

Supplemental Figure 3

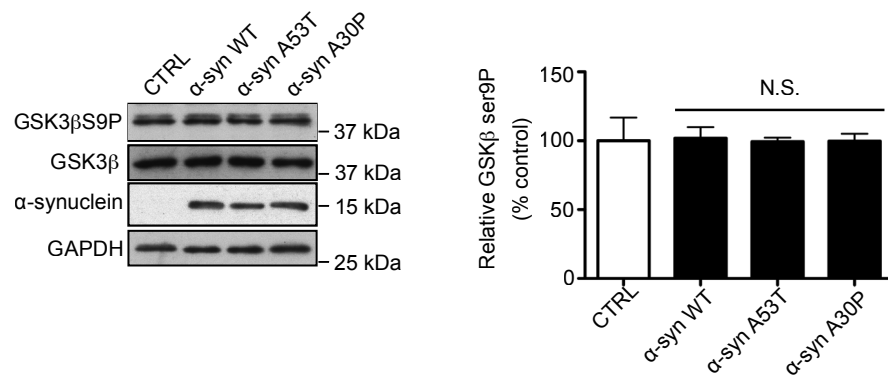

Supplement: Supplementary file 3 — Supplemental Fig. 3 Overexpression of α-synuclein does not activate GSK3β. Immunoblots of SH-SY5Y cells expressing control empty vector, wild-type α-synuclein, α-synucleinA53T or α-synucleinA30P probed for inactive GSK3β phosphorylated on serine-9 (GSK3β-P), total GSK3β, α-synuclein and GAPDH as a loading control. Molecular masses in kD are shown on the right. Bar chart shows GSK3β serine-9 phosphorylation signals normalized to controls. Data were analysed by one-way ANOVA. N = 4; error bars are SEM, N.S. not significant (PDF 152 kb) [file 401_2017_1704_MOESM3_ESM.pdf]
